# Revealing Principal Components, Patterns, and Structural Gaps in Health Security among High-Income Countries: A Comparative Analysis Using PCA and a Multi-Scenario Clustering Approach

**DOI:** 10.12688/f1000research.168082.2

**Published:** 2025-09-11

**Authors:** Adel A. Nasser, Mijahed Nasser Aljober, Abed Saif Ahmed Alghawli, Amani A. K. Essayed

**Affiliations:** 1 Information Systems and Computer Science, Sa’adah University, Sa'adah, Yemen; 2 Artiﬁcial Intelligence, Modern Specialized University, Sana'a, Yemen; 3 Computer Science, Prince Sattam bin Abdulaziz University, Al Kharj, Riyadh Province, Saudi Arabia

**Keywords:** health security; high-income countries; Principal Component Analysis clustering; K-means; influential indicators

## Abstract

**Objectives:**

The COVID-19 pandemic highlighted significant weaknesses in health security systems, even in high-income countries (HICs), underscoring the necessity for a more nuanced understanding of their distinct strengths and vulnerabilities. Existing research often offers broad evaluations and fails to capture the complex internal dynamics of health-security performance. This study seeks to fill this gap by identifying the latent factors that define health security capacities in HICs and clustering countries based on these factors.

**Methods:**

A multistage analytical framework was employed based on the 2017–2021 Global Health Security Index (GHSI) dataset. Initially, Principal Component Analysis (PCA) with varimax rotation was applied to the 37 GHSI indicators to reduce dimensionality and reveal latent structures within the data. This process identified nine principal components for the subsequent analysis. Subsequently, K-means clustering was utilized under three methodological scenarios: using countries’ average scores across the nine extracted components, based on 13 high-loading indicators from the first principal component, and using aggregated scores across the six original GHSI categories. This design facilitated a comprehensive comparison of the clustering outcomes across different data representations.

**Results:**

Analysis found nine components that together explained 74.50% of the total differences, with the first component—"Foundational Capacity, Regulations, Resilience, and Prevention-Detection Systems"—making up 37.62% of that total. Together, the first three components explained 51.81% of the total variance. Clustering across all three scenarios categorized high-income countries into four levels, revealing significant disparities. Nauru, the Cook Islands, and Palau consistently ranked lowest, highlighting critical gaps in foundational capacities and systemic readiness despite their high-income status. This study shows that wealth alone does not ensure preparedness, revealing distinct performance patterns and weaknesses across countries.

**Conclusion:**

The findings underscore the need for tailored policies, multi-method evaluations, and sustained global cooperation to enhance resilience and guide investments in national and global health security.

**Glossary**
AMR: Antimicrobial Resistance
COVID-19: Coronavirus Disease 2019
EHRs: Electronic Health Records
GHSI: Global Health Security Index
HICs: High-Income Countries
JEE: Joint External Evaluation
KMO: Kaiser-Meyer-Olkin
LMICs: Low- and Middle-Income Countries
MCDM: Multi-Criteria Decision-Making
PCA: Principal Component Analysis
PVS: Performance of Veterinary Services
SSE: Sum of Squared Errors
UHC: Universal Health Coverage

## 1. Introduction

The profound and far-reaching impact of recent global pandemics, particularly COVID-19, has shifted the discourse on health security from a theoretical issue to an urgent global priority. In our highly interconnected world, localized outbreaks can swiftly escalate into global crises, disrupting economies, destabilizing societies, and claiming numerous lives. ^1^ These developments underscore the necessity of strengthening health security systems, not only as a national concern but also as a critical global obligation to protect human rights. The effectiveness of these systems, especially in high-income regions, hinges on their ability to swiftly detect and respond to emerging health threats. This requires robust cross-border collaboration, resilient healthcare infrastructure, equitable resource distribution, and the implementation of coordinated, data-driven strategies supported by sound policy interventions. ^2, 3^

High-income countries (HICs), often regarded as leaders in health preparedness due to their advanced healthcare infrastructure, scientific capacity, and substantial financial resources, encountered significant shortcomings during the pandemic. ^4– 6^ These unexpected gaps between perceived preparedness and actual response performance reveal systemic weaknesses. ^7^ Despite the presence of robust frameworks and considerable investments, there remains a lack of detailed insight into the specific strengths and vulnerabilities that define health security in HICs. ^6, 7^ These gaps highlight the urgent need for a more nuanced and evidence-based understanding of how well-prepared even the most resource-rich nations truly are, as well as for informed decision-making grounded in that evidence. ^2, 3, 8, 9^

Existing research on global health security often adopts either broad aggregate assessments or focuses narrowly on specific dimensions of preparedness. Numerous studies have evaluated the Global Health Security Index (GHSI) in terms of its utility for assessing countries’ readiness for biological threats, ^10^ its statistical correlation with Universal Health Coverage (UHC), ^11^ and the role of community health workers in strengthening system resilience. ^12^ Other investigations have explored the impact of international support programs, ^13^ political and socioeconomic contexts shaping health security financing, ^14^ and limited progress in global preparedness despite regular updates to GHSI scores. ^15^ The practical applications and initiatives derived from the GHSI for policy and decision-making have also been examined, ^16– 18^ along with critical questions about why some highly developed nations underperformed during the COVID-19 pandemic. ^19^ Additionally, researchers have studied the GHSI’s association with excess mortality due to COVID-19, ^20^ the cultural and socioeconomic contexts influencing pandemic outcomes, ^21^ and its integration with digital security dimensions such as cybersecurity metrics. ^22^ While these studies provide valuable macro-level insights, they often fail to capture the nuanced internal dynamics and hidden variables that explain disparities in health security performance, especially within ostensibly homogeneous groups, such as high-income countries (HICs). ^9, 23^

The GHSI offers a robust framework comprising six core domains: prevention, detection and reporting, rapid response, health system, compliance with international norms, and risk environment. ^7^ However, using all 37 indicators or relying solely on aggregated domain scores may obscure critical interdependencies, introduce redundancy, and complicate result interpretation. Composite group scores can mask the influence of key sub-indicators, and full-indicator approaches often suffer from statistical inefficiencies, potentially diluting meaningful patterns and leading to misleading conclusions. ^2, 3, 8, 9^

Considering the utility and advantages of Multi-Criteria Decision-Making (MCDM) models, and machine learning clustering approaches as well as their integration in addressing complex decision-making problems across diverse domains, ^24– 35^ several studies have introduced advanced MCDM and clustering methods to overcome these limitations. For instance, a study using MAIRCA on EU member states found a strong alignment between GHSI rankings and those derived from most MCDM methods, except for MAUT, suggesting a general agreement on performance patterns. ^36^ Another study employing MCDM, and clustering techniques identified inconsistencies between GHSI rankings and actual COVID-19 outcomes, underscoring the need to revise the index’s predictive framework. ^37^ Additional studies ^2, 3, 8, 9, 23^ proposed integrated MCDM weighting and ranking models—combined with k-means clustering—to assess health security performance in Africa, the Eastern Mediterranean, Western Asia, EU, and Non-EU Europe, using 2019 and 2021 data, as well as aggregated scores from 2017 to 2021.

These contributions offer detailed regional analyses, helping establish priorities, track performance trends, explore patterns, and enable meaningful cross-regional comparisons. However, none have specifically targeted the internal patterning and latent structural components of health security in high-income countries. Moreover, prior research has predominantly relied on either the full set of GHSI indicators or the aggregated scores of the six main domains. While comprehensive in appearance, such approaches may hinder analytical precision and conceal the underlying drivers of performance.

What remains lacking is a focused effort to systematically uncover the latent factors that define health security capacities in HICs and to understand how these components collectively shape the preparedness outcomes. Addressing this analytical gap requires moving beyond descriptive statistics and adopting advanced multivariate techniques to extract dominant dimensions, reduce redundancy, and generate actionable insights that support more targeted, evidence-based interventions. Specifically, the judicious application of dimensionality reduction techniques, such as Principal Component Analysis (PCA), followed by sophisticated clustering analysis, offers a powerful avenue to reveal distinct patterns of health security performance that are not readily discernible through conventional methods. This integrated approach facilitates a more granular understanding of how diverse health security indicators coalesce into broader, interpretable dimensions, and how countries naturally group based on these underlying structures.

Drawing from the reviewed literature and identified limitations in current methodologies, this study posits the following hypotheses: (1) high-income countries will display considerable variation in health security capacity despite their similar economic standing; (2) clustering based on PCA will uncover latent structures and groupings that significantly diverge from the original GHSI categories; and (3) employing dominant principal components will yield more precise insights into foundational health security capacities than aggregate indicators. This study was meticulously designed to explore principal components, reveal performance patterns, and identify structural gaps in health security among high-income countries. Specifically, it aims to achieve two core objectives: first, to empirically extract the latent dimensions underpinning health security through principal component analysis, and second, to systematically classify countries based on their alignment with these dimensions, thereby uncovering critical disparities and structural weaknesses in their health security performance.

To achieve these objectives, this study employs a multistage methodological approach. Initially, it utilized the 2021 GHSI Data Model and Report, meticulously incorporating revised 2019 scores to construct a robust and consistent dataset. ^38^ The deliberate focus on HICs is justified by their pivotal role in global health security and the imperative to dissect their nuanced capabilities. Subsequently, Principal Component Analysis (PCA) with varimax rotation was systematically applied to the 37 GHSI indicators. This method was strategically chosen for its proven efficacy in reducing high-dimensional data into a more manageable set of uncorrelated principal components, thereby precisely identifying the most influential factors shaping health security. The PCA results not only confirm the inherent suitability of the dataset for such rigorous analysis but also unveil the underlying components that collectively explain the majority of the variance in health security performance.

Finally, following the precise identification of these principal components, a comprehensive clustering analysis, specifically K-means clustering, was performed. This clustering is executed under three distinct, yet interconnected, cases: (1) based on countries’ average GHSI performance across the nine extracted principal components; (2) based on the 13 most influential indicators derived from the dominant first principal component; and (3) as a crucial baseline comparison, utilizing country-level GHSI scores across the six original GHSI categories. The Elbow Method was rigorously applied to determine the optimal number of clusters, ensuring a judicious balance between parsimony and explanatory power. This innovative multi-case clustering approach facilitates a robust comparative assessment of national performance levels and precise identification of consistent groupings, ultimately yielding a profoundly nuanced understanding of health security patterns.

The contributions of this study are both multifaceted and significant. Theoretically, it enhances our understanding of health security by revealing latent dimensions that influence preparedness in high-income countries, moving beyond the traditional categorical assessments.

The methodology of this study demonstrates the effectiveness of integrating principal component analysis (PCA) with multi-case clustering. This combination reveals detailed country profiles and underscores performance disparities that might be hidden when relying solely on a single analytical framework. This comprehensive multi-method approach enables policymakers to triangulate evidence, prioritize interventions based on systemic influences and identified categorical gaps, and circumvent the limitations inherent in any singular assessment method.

These findings provide invaluable and actionable insights for global health policymakers and stakeholders. By identifying specific strengths and vulnerabilities within different HIC clusters, this study aids in the development of tailored policy recommendations and targeted interventions. This paper highlights the essential role of international partnerships and robust funding mechanisms, especially for lower-performing clusters, in overcoming persistent structural barriers. Ultimately, this study seeks to foster a more nuanced understanding of global health security preparedness, enabling more informed policy evaluation and strategic planning in an increasingly interconnected and volatile world. These insights are crucial for strengthening national resilience and safeguarding global public health against future threats, emphasizing that high-income status alone does not ensure preparedness but requires strategic coherence, operational excellence, and unwavering commitment across all pillars of preparedness and response. Thus, this study serves as a vital resource for global health policymakers, researchers, and practitioners dedicated to enhancing health security.

## 2. Methods

This study adopted a multistage methodological approach to evaluate health security performance in high-income countries. It begins by utilizing the 2021 Global Health Security Index (GHSI) Data Model and Report, incorporating revised 2019 scores to create a robust dataset. Next, Principal Component Analysis (PCA) with varimax rotation was applied to the 37 GHSI indicators to identify the most influential factors shaping health security. Finally, a comprehensive clustering analysis was conducted under three distinct scenarios: based on countries’ average GHSI performance across nine extracted principal components, using the 13 most influential indicators from the dominant first principal component, and employing country-level GHSI scores across the six original GHSI categories. The Elbow Method was used to determine the optimal number of clusters, ensuring a balance between parsimony and explanatory power.

### 2.1 Study design, sample, and data collection

*2.1.1 **Global Health Security Index (GHSI) as the analytical framework***

This investigation rigorously utilized the Global Health Security Index (GHSI) as its foundational analytical framework. The GHSI, a publicly accessible and comprehensive assessment tool, systematically categorizes 37 distinct sub-indicators across six core domains, each addressing a critical aspect of global health security (GHSI, 2021; Global Health Security Index Data Model and Report, 2021). ^38^ These domains are outlined as follows ^38^:

1. Prevention of the Emergence or Release of Pathogens: This domain evaluates national capacities in areas such as combating antimicrobial resistance, managing zoonotic disease threats, ensuring laboratory biosafety protocols, and implementing robust immunization programs.
2. Early Detection and Reporting for Epidemics of Potential International Concern: This category assesses the robustness of national laboratory systems, real-time surveillance capabilities, proficiency of the epidemiology workforce, and degree of data integration across human, animal, and environmental health sectors.
3. Rapid Response to and Mitigation of the Spread of an Epidemic: This domain emphasizes national preparedness and emergency response planning, inter-sectoral coordination between public health and security entities, the efficacy of risk communication strategies, and the availability of critical communication infrastructure.
4. Sufficient and Robust Health Sector to Treat the Sick and Protect Health Workers: This category scrutinizes the capacity of national healthcare systems, availability of essential medical countermeasures and adequately trained personnel, accessibility of healthcare services, and implementation of stringent infection prevention and control practices.
5. Commitments to Improving National Capacity, Financing, and Adherence to Norms: This domain encompasses indicators related to compliance with International Health Regulations (IHR), active participation in cross-border agreements and international health initiatives, the execution of external evaluations such as the Joint External Evaluation (JEE) and the Performance of Veterinary Services (PVS), and the establishment of sustainable financing mechanisms for health security.
6. Overall Risk Environment and Country Vulnerability to Biological Threats: This final category considers overarching factors such as political stability, socioeconomic resilience, robustness of critical infrastructure, prevailing environmental challenges, and inherent public health vulnerabilities that collectively influence a nation’s capacity to effectively manage biological threats.

*2.1.2 **Rationale for GHSI selection and methodological considerations***

To enhance the methodological rigor and transparency of this study, it is essential to clarify the rationale for selecting the GHSI as the primary source of data. The GHSI is widely recognized for its comprehensive scope and ability to provide standardized cross-national assessments of health security capabilities. ^2, 3^ Its indicators are meticulously developed through a multi-stakeholder process, incorporating expertise from diverse fields, thereby contributing to its robustness and contemporary relevance. ^7– 9^ The decision to compute a composite score by averaging the 2019 and 2021 GHSI scores was methodologically sound. This approach effectively mitigates the impact of potential year-to-year variability and transient reporting anomalies, thereby yielding a more stable and reliable representation of a country’s health security posture over time. ^23^ This methodological refinement enhanced the reliability of the dataset for subsequent analytical procedures.

*2.1.3 **Focus on High-Income Countries (HICs) and data specifics***

This study specifically examines high-income countries (HICs) to investigate patterns and disparities in health security performance within economically advanced contexts. The intentional focus on HICs is based on the necessity to explore health security in environments where financial and infrastructural constraints are relatively minimal. This approach allows for a clearer investigation of the strategic, policy-related, and systemic determinants influencing preparedness. Previous research has often categorized nations based on geographical regions, a practice that can inadvertently obscure variations in capacity that correlate more strongly with economic status than geographical location. In contrast, this study employs the World Bank’s four-tier income classification system—comprising low, lower-middle, upper-middle, and high-income categories—as a more analytically relevant framework for the study.

Although upper-middle-income countries could provide additional comparative insights, they were excluded from this study to maintain analytical consistency. By focusing exclusively on high-income countries (HICs), the analysis allows for a clearer interpretation of variations arising from governance, infrastructure, and policy differences without the confounding effects of financial or resource constraints, which are more prevalent in lower income categories. This focus also facilitates the assessment of whether elevated economic development consistently translates into superior health security outcomes and provides a robust basis for identifying internal gaps or inefficiencies that may persist despite abundant resources in the country. Although HICs share common macroeconomic characteristics, they display considerable heterogeneity in governance structures, healthcare delivery models, and public health priorities. ^40^

From a data perspective, this study exclusively utilized the 2021 GHSI data report, meticulously incorporating revised 2019 scores to ensure comprehensive data completeness and consistency (GHSI, 2021). To effectively mitigate the influence of short-term fluctuations and reporting inconsistencies, a composite score for each of the 37 indicators was systematically calculated by averaging the corresponding values from the 2019 and 2021 GHSI Report. This methodological approach yields a more stable and directly comparable representation of national health security capacity across the four-year period spanning 2017–2021, aligning with established practices that enhance data stability in longitudinal assessments.

### 2.2 Sample Description: High-Income Countries Analyzed

This study concentrated solely on high-income countries (HICs) as defined by the World Bank’s income classification system. According to this framework, countries are divided into four income groups—low, lower-middle, upper-middle, and high income —based on the gross national income (GNI) per capita. For this analysis, only nations classified as high-income during the study period were included in the analysis. A total of 59 high-income countries were examined ^38^: Andorra, Antigua and Barbuda, Australia, Austria, Bahamas, Bahrain, Barbados, Belgium, Brunei, Canada, Chile, Cook Islands, Croatia, Cyprus, Czech Republic, Denmark, Estonia, Finland, France, Germany, Greece, Hungary, Iceland, Ireland, Israel, Italy, Japan, Kuwait, Latvia, Liechtenstein, Lithuania, Luxembourg, Malta, Monaco, Nauru, Netherlands, New Zealand, Norway, Oman, Palau, Poland, Portugal, Qatar, San Marino, Saudi Arabia, Seychelles, Singapore, Slovakia, Slovenia, South Korea, Spain, St. Kitts and Nevis, Sweden, Switzerland, Trinidad and Tobago, United Arab Emirates, United Kingdom, United States of America, and Uruguay.

The deliberate focus on HICs is methodologically significant for two reasons. First, it ensured analytical consistency by examining countries operating under broadly similar macroeconomic conditions, where resource and infrastructure constraints are relatively minimal. Second, it allowed for an investigation into whether higher economic capacity reliably translates into stronger health security systems or whether systemic vulnerabilities persist despite greater financial and technological resources.

### 2.3 Principal Component Analysis with Varimax Rotation for factor identification and variable selection

Principal Component Analysis (PCA) is a widely used unsupervised machine learning and statistical method primarily employed for dimensionality reduction and feature extraction. ^41^ Its main goal is to transform high-dimensional datasets into a smaller set of uncorrelated variables, known as principal components, while maximizing the retention of the original data variance. ^42^ PCA is a fundamental tool in various scientific and engineering fields, including data analysis, pattern recognition, and statistical learning. ^43^ The benefits of PCA include enhanced computational efficiency, ^44^ improved interpretability of complex datasets, ^45^ and the ability to reveal latent structures within intricate data landscapes. ^46, 47^

A key strength of PCA is its capacity to orthogonalize correlated variables into a new set of principal components. ^48^ This transformation projects data onto new axes that sequentially capture the maximum possible variance. ^49^ Each principal component is mathematically defined as a linear combination of the original variables with coefficients derived from the eigenvectors of the covariance or correlation matrix. ^47^ The corresponding eigenvalues quantify the proportion of the variance explained by each component. ^47, 49^ Components are retained based on their explanatory power, typically following the Kaiser criterion, which requires an eigenvalue greater than 1. ^46^ In this study, PCA was applied to the standardized correlation matrix of 37 Global Health Security (GHS) indicators, specifically for high-income countries. Before factor extraction, the dataset’s suitability for PCA was rigorously confirmed through the Kaiser-Meyer-Olkin (KMO) measure of sampling adequacy and Bartlett’s test of sphericity. Both statistical tests met conventional thresholds (KMO > 0.6; Bartlett’s p < 0.05), affirming the data’s factorability. ^50, 51^

After factor extraction, varimax rotation, a commonly used orthogonal rotation technique, was employed to improve the interpretability of the component structure. ^51^ This method redistributes the variance among the factors and maximizes the variance of the squared loadings, thereby clarifying the associations between the original variables and the derived components. Indicators with absolute loadings ≥ 0.5 were deemed statistically significant and representative of their associated latent constructs. The rotated component matrix subsequently categorized all 37 GHS indicators into nine distinct and interpretable components, each hypothesized to represent a latent domain of national health security capacity. The first component, labeled ‘Foundational Capacity, Regulations, Resilience, and Prevention—Detection Systems,’ emerged as the most influential, characterized by 13 indicators with strong, negative loadings. These indicators collectively reflect the core national capacities in surveillance, biosafety, clinical response, supply chain management, and adherence to international commitments. While all components and their associated variables were retained for comprehensive thematic interpretation, the 13 high-loading variables from Component 1 were specifically identified as the most influential subset and were subsequently selected for the ensuing clustering analysis.

This selection was based on their dominant contribution to the total explained variance and strong conceptual coherence. The outcomes of this analytical stage are twofold:

• A comprehensive classification of all 37 GHS indicators into nine principal components, enabling nuanced thematic interpretation of health security domains, considering.
• The judicious selection of 13 key indicators from the most dominant component, which will serve as the primary input for clustering high-income countries based on their shared health security performance characteristics.

The following outlines the computational steps of the PCA–Varimax rotation process implemented in this study.

Let $X={\left[x_{\mathrm{ij}}\right]}_{\mathrm{mxn}}\;$ be the decision matrix where i = 1,2,…,m, represents high-income countries, and j = 1,2, …, n represents the GHS indicators (37 indicators). The steps of the PCA method are as follows ^51, 52^:

• Step 1: Construct the Health Security Covariance Matrix

Compute the Covariance matrix $C={\left[c_{\mathrm{jk}}\right]}_{\mathrm{mxn}}$ , where each element $c_{\mathrm{jk}}$ measures the covariance between the j-th and k-th indicators:

$$c_{\mathrm{jk}}=\frac{\sum_{i=1}^{m}\left(x_{\mathrm{ij}}-\bar{x_{j}}\right)\left(x_{\mathrm{ik}}-\bar{x_{k}}\right)}{m-1}$$ (1)

Here, $\bar{x_{j}}$ and $\bar{x_{k}}$ are the mean values of indicators j and k across all countries.

• Step 2: Assess Dataset Suitability

Perform the Kaiser-Meyer-Olkin (KMO) and Bartlett’s test of sphericity. The KMO value should exceed 0.6, and Bartlett’s test should be significant at p < 0.05 to proceed with PCA. ^50, 51^

• Step 3: Factor Extraction Using PCA

Determine the eigenvalues λ and eigenvectors ϑ of the covariance matrix C by solving the characteristic Equation (2), and the eigenvalue Equation (3):

det(C−λI)=0 (2)

(C−λI)ϑ=0 (3)

Where I is the identity matrix. Each eigenvalue $\lambda_{i}\;$ quantifies the variance explained by the corresponding eigenvector $\;\vartheta_{i}$ . Retain factors with eigenvalues greater than 1, following the Kaiser criterion. ^51^

• Step 4: Apply Varimax Rotation

Enhance interpretability by applying orthogonal Varimax rotation to the extracted components. A variable is considered significantly associated with a factor if its loading is ≥0.5 in absolute value.

• Step 5: Interpret Rotated Factors and Loadings Classify indicators based on rotated loadings: Interpret each factor according to the variables associated with it and identify the dominant component for further analysis.

To further improve the interpretability of the extracted components, Varimax rotation was applied subsequent to the initial Principal Component Analysis (PCA). While PCA is highly effective in achieving dimensionality reduction, the resultant components can often be challenging to interpret due to potential moderate correlations with a broad spectrum of original variables. To address this, Varimax rotation, an orthogonal rotation technique, was employed to maximize the variance of squared loadings within each component. This method facilitates the creation of a simpler and more interpretable factor structure, wherein each component is more distinctly associated with a limited number of variables, thereby enhancing the clarity of the underlying latent constructs. ^46^

An absolute loading threshold of 0.5 was established to identify variables contributing significantly to each component. This threshold is a widely accepted practice in factor analysis and ensures that only variables with meaningful associations are retained for subsequent analysis. The application of this cutoff enabled the delineation of well-defined components, which was crucial for the subsequent clustering analysis and thematic classification of health security domains. Although alternative rotation methods permit correlated factors, Varimax was specifically chosen for its capacity to produce clearly interpretable and uncorrelated factors, a characteristic particularly valuable in exploratory analyses. While acknowledging that this orthogonality assumption might simplify the complex interrelations among health system variables, the resultant gain in interpretability was deemed a justified trade-off within the context of this study. This methodological choice and its implications were carefully considered to ensure analytical transparency and rigor throughout the factor-based clustering process.

### 2.4 Clustering approach for identifying health security performance patterns in high-income countries

After conducting dimensionality reduction and variable selection through Principal Component Analysis (PCA) with Varimax rotation, a clustering analysis was carried out to identify distinct patterns of health security performance among high-income countries. Clustering, a widely used unsupervised machine learning technique, organizes unlabeled data into meaningful groups based on inherent similarities. ^2, 8^ Unlike supervised learning models that depend on labeled data, clustering algorithms uncover hidden structures and natural groupings within datasets, making them particularly valuable for exploratory analysis and hypothesis generation. ^53^ K-means clustering was chosen for this study due to its computational efficiency, simplicity, and proven effectiveness in identifying distinct, non-overlapping clusters within quantitative datasets. ^2, 3, 9, 23^ This choice aligns with the study’s goal of categorizing countries into discrete performance tiers based on their health security profiles. The algorithm minimizes within-cluster variance through an iterative process that involves randomly initializing k centroids within the data space, assigning each data point to the nearest centroid to form clusters, updating centroids as the mean of all points in each cluster, and repeating the assignment-update cycle until convergence is achieved or a maximum number of iterations is reached. To explore various grouping patterns and provide a comprehensive assessment, three separate clustering experiments were conducted, each guided by a distinct rationale. The first case involved principal component-based clustering using the nine principal components extracted from PCA. In this approach, countries were clustered based on their average Global Health Security Index (GHSI) performance across groups of indicators with strong factor loadings (≥0.5) under each of the nine components. This method offered a holistic representation of health security performance by integrating multiple latent dimensions. The second case also employed a PCA-based approach but focused solely on the 13 indicators with strong loadings on the first principal component, which was identified as the most influential in explaining variance across the dataset. This allowed for the identification of clustering patterns shaped by the dominant factor in national health security capacity. The third case served as a baseline comparison, clustering countries using their scores across the six original health security categories defined by the GHSI. This benchmark case enabled evaluation of how the PCA-based approaches aligned with or diverged from the established framework.

Before implementing clustering, the relevant country-level scores for each case were extracted from the original dataset. Since all indicators were already on a uniform scale ranging from 0 to 100, additional normalization techniques such as z-score standardization or min-max scaling were unnecessary. This decision preserved the original interpretability of the scores while avoiding disproportionate influence from variables with larger numerical ranges.

To determine the optimal number of clusters (k), the Elbow Method was applied in each case. ^8^ This approach involved plotting the total within-cluster Sum of Squared Errors (SSE) against increasing values of k and identifying the point where the reduction in SSE began to plateau. ^8^ In all three clustering scenarios, a sharp decline in SSE was observed from k = 1 to k = 2, followed by a more gradual decrease, with the elbow consistently appearing at k = 4 or 5. Consequently, k = 4 was selected for all clustering procedures to ensure parsimony and explanatory strength.

In the final stage of analysis, K-means clustering was applied to group high-income countries into distinct performance categories based on their scores derived from the principal component analysis (PCA). The objective of this step was to identify underlying patterns in national health security performance by partitioning countries into four clusters with similar profiles across the extracted components. The first cluster represented the high-performing group, comprising countries with strong national health security systems that excelled in governance, infrastructure, and emergency preparedness. The second cluster consisted of countries with moderate-to-high performance, generally demonstrating solid capacity but requiring targeted improvements in specific areas. The third cluster included countries with low-to-moderate performance, characterized by below-average capacities and ongoing challenges in key areas, although some strengths were evident. Finally, the fourth cluster reflected countries of critical concern, which consistently exhibited low scores across core indicators.

This four-tier classification has practical value for global health policy, as it facilitates differentiated strategies for risk mitigation, resource allocation, and international cooperation. Moreover, it enhances the interpretability of the PCA-derived components and supports a structured framework for cross-country comparisons and prioritizing interventions.

K-means clustering was chosen specifically for its compatibility with medium-sized quantitative datasets, ^54^ such as the one used in this study, comprising 59 high-income countries and a reduced set of indicators. Its speed, interpretability, and ability to minimize within-cluster variance make it well suited for exploratory classification tasks. However, the method’s assumption of spherical and equally sized clusters may not always align with real-world data. The consistent emergence of well-defined four-cluster structures across all three experimental cases provides strong empirical support for the appropriateness of K-means in this context.

The decision to employ three distinct clustering strategies is a methodological strength of this study, enabling a multi-perspective analysis of health security patterns and enhancing both the depth and credibility of the findings. Clustering based on all nine principal components offered the most comprehensive view by incorporating a full spectrum of latent dimensions. In contrast, clustering based on the first principal component provides focused insight into countries’ foundational health security capabilities. Clustering using the original GHSI categories served as an important reference point, allowing for the validation and benchmarking of the PCA-based models.

## 3. Results

The results highlight several policy-relevant insights that are critical for strengthening global health security in high-income countries (HICs). First, the analysis confirms that high economic status does not equate to strong preparedness: several small island nations—including Nauru, the Cook Islands, and Palau—consistently clustered at the lowest performance levels despite their high income classification. Second, while leading countries such as the United States, Australia, and several European nations exhibit robust foundational capacities, they also show persistent weaknesses in operational readiness, workforce capacity, and case-based investigation—gaps that could undermine timely responses to crises. Third, the clustering analysis revealed a distinct stratification into four performance tiers, underscoring systemic disparities even among advanced economies and pointing to the need for targeted investments in surveillance, biosafety, financing mechanisms, and emergency response operations—areas repeatedly identified as common weaknesses across clusters. Finally, the findings emphasize that methodological choice matters: whether clustering is based on PCA-derived components, the original GHSI categories, or MCDM approaches, the resulting groupings and priority areas differ, underscoring the importance of using multiple analytical lenses when informing policy decisions.

### 3.1 Principal Component Analysis with Varimax Rotation: Factor extraction and indicator structuring

The principal component analysis (PCA) results confirmed the dataset’s suitability for dimensionality reduction and latent structure identification (see Supplementary File: Principal Component Extraction). ^39^ Bartlett’s test of sphericity was highly significant (χ ^2^ = 1614.8, df = 36, p < 0.01), indicating sufficient intercorrelations among the indicators to justify factor analysis.

**Figure 1.**
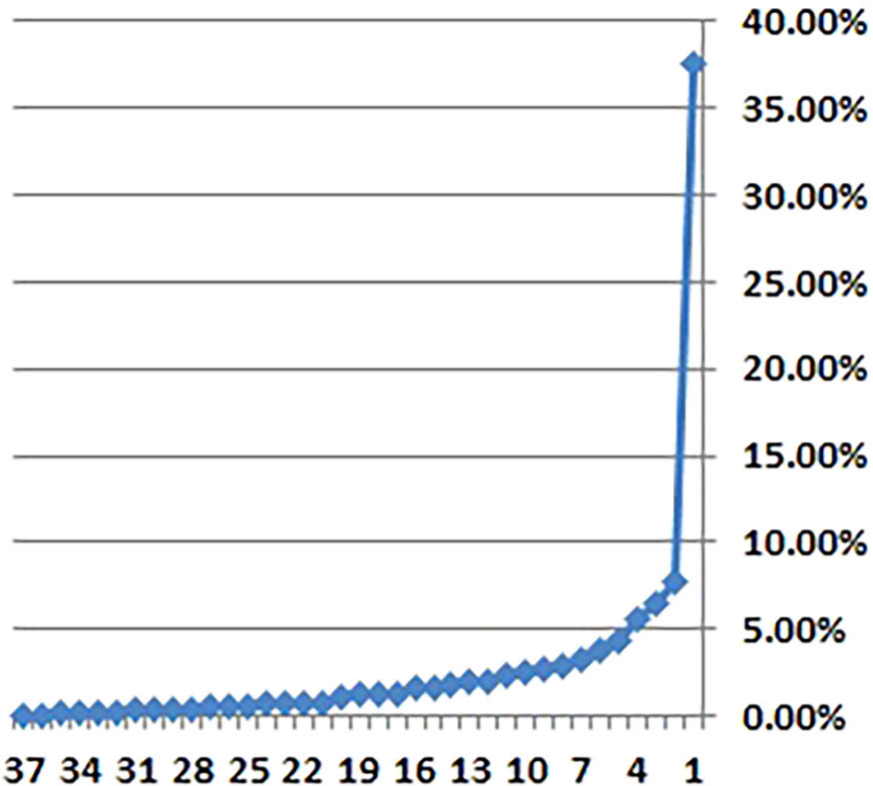
Scree Plot. Figure 1 provides a graphical representation of the eigenvalues of the principal components, used to determine the number of factors to retain in the analysis.

The Kaiser-Meyer-Olkin (KMO) measure of sampling adequacy was 0.761 for the overall dataset, which is considered above average, further validating the suitability of PCA (see Supplementary File: Bartlett’s and KMO tests). ^39^ However, two individual indicators—political and security risk (KMO = 0.393) and healthcare access (KMO = 0.444)—fell below the acceptable threshold of 0.5, suggesting a weaker representation in the factor structure. Despite these exceptions, the overall test results supported the PCA. Based on Kaiser’s criterion (eigenvalue >1) and the scree plot ( Figure 1), nine principal components were retained for further analysis.

These nine components collectively accounted for 74.50% of the total variance, with the first component explaining 37.62%. The first three components cumulatively captured 51.81% of the total variance, indicating that they reflected a substantial portion of the overall variation in the data.

Table 1 presents the eigenvalues and explained variance for each component, while Table 2 details the factor loadings (≥|0.5|) used to interpret and label the components. The rotated component matrix revealed meaningful groupings of indicators, confirming the multidimensional nature of health security performance in high-income countries (HICs). Specifically, key prevention-related indicators, such as antimicrobial resistance (1.1), biosecurity (1.3), biosafety (1.4), dual-use research and responsible science (1.5), and immunization (1.6), were distributed across three distinct components, reflecting their diverse yet interrelated roles. Similarly, indicators pertaining to prevention and detection systems loaded onto four components, underscoring their unique contributions to health security readiness. These results highlight the complexity and interconnectedness of health security domains, providing a robust foundation for subsequent clustering and comparative analyses across HICs.

**Table 1.** Principal health security components in HICs.

PCA	Components	Eigenvalue	% of variance	Cumulative % of variance
1	Foundational Capacity, Regulations, Resilience, and Prevention-Detection Systems	13.9181	37.62%	37.62%
2	Laboratory supply chains and Cross-Border Health Agreements	2.87941	7.78%	45.40%
3	Political Stability and Infrastructure Adequacy	2.373703	6.42%	51.81%
4	Communication Access and Immunization Coverage	2.070713	5.60%	57.41%
5	Operational Readiness and Financial Support	1.631398	4.41%	61.82%
6	Workforce, Communication, and Research	1.399973	3.78%	65.60%
7	Healthcare access	1.22277	3.30%	68.91%
8	Case-based investigation	1.067205	2.88%	71.79%
9	Biological Data Sharing and Emergency Response Preparedness	1.001272	2.71%	74.50%

Table 1 presents the nine principal components of health security in high-income countries, along with their corresponding eigenvalues and the percentage of variance explained by each component.

**Table 2.** Classification of health security components with significant factor loadings (the absolute value of the correlation greater than or equal to 0.5).

PCA	Components	Indicators	Positive loadings	Negative loadings
1	Foundational Capacity, Regulations, Resilience, and Prevention-Detection Systems	1.1) Antimicrobial resistance (AMR)		-0.66451
		1.3) Biosecurity		-0.79325
		1.4) Biosafety		-0.73178
		2.1) Laboratory systems strength and quality		-0.84909
		2.3) Real-time surveillance and reporting		-0.60787
		2.4) Surveillance data accessibility and transparency		-0.87513
		3.4) Linking public health and security authorities		-0.77677
		4.1) Health capacity in clinics, hospitals and community care centers		-0.63604
		4.2) Supply chain for health system and healthcare workers		-0.72928
		4.6) Infection control practices		-0.73186
		4.7) Capacity to test and approve new medical countermeasures		-0.7894
		5.3) International commitments		-0.81003
		6.2) Socio-economic resilience		-0.54874
2	Laboratory supply chains and Cross-Border Health Agreements	2.2) Laboratory supply chains	0.601126	
		5.2) Cross-border agreements on public health and animal health emergency response		-0.68584
3	Political Stability and Infrastructure Adequacy	6.1) Political and security risk	0.920661	
		6.3) Infrastructure adequacy	0.65982	
4	Communication Access and Immunization Coverage	3.6) Access to communications infrastructure		-0.83358
		1.6) Immunization		-0.50373
5	Operational Readiness and Financial Support	3.3) Emergency response operation		-0.66606
		5.5) Financing		-0.76973
6	Workforce, Communication, and Research	5.4) JEE and PVS	0.809392	
		4.5) Communications with healthcare workers during a public health emergency	0.550554	
		2.6) Epidemiology workforce	0.60941	
		1.5) Dual-use research and culture of responsible science	0.692359	
7	Healthcare access	4.4) Healthcare access	0.886682	
8	Case-based investigation	2.5) Case-based investigation		-0.64371
9	Biological Data Sharing and Emergency Response Preparedness	3.2) Exercising response plans	0.636941	
		5.6) Commitment to sharing of genetic & biological data & specimens		-0.57598

Table 2 details the classification of health security indicators into these nine components, based on their significant factor loadings from the Principal Component Analysis.

Furthermore, the distribution of variables among the principal components ( Table 2) revealed several distinct thematic clusters. One such cluster highlights readiness enablers, where variables such as emergency response operations and sustainable financing emerge as closely interlinked operational prerequisites—financial commitment directly underpins the capacity for effective emergency mobilization. These indicators share similar characteristics in terms of resource dependency and implementation-oriented functions, exemplifying a strong association in a common operational domain. Similarly, variables such as health communication with workers, the epidemiology workforce, and dual-use research form another coherent cluster, emphasizing a unique health security dimension centered on institutional capacity and scientific governance. Another notable grouping involved the co-loading of exercise response plans and sharing genetic and biological data, collectively representing a dimension of information-based preparedness. While the former emphasizes internal preparedness through system rehearsals, the latter reflects external transparency and global cooperation. Conceptually, both indicators underscore the importance of readiness achieved through knowledge exchange and coordinated information management. An especially interesting finding is that variable 4.4 (healthcare access) loads independently of a standalone component. This suggests that access to healthcare is conceptually and statistically distinct from other dimensions of health security. While systemic infrastructure and foundational capacities are essential, physical access to care operates on a separate axis, often reflecting equity, service availability, and population reach rather than system-wide functionality. Additional indicators—2.5 (case-based investigation), 3.2 (exercising response plans), 5.6 (sharing genetic and biological data), and 5.2 (cross-border health agreements)—appear in separate clusters, indicating their independent influence on health security performance. These variables represent specialized functions, including targeted surveillance, scenario-based simulations, international data-sharing obligations, and global health diplomacy. Although they may not align closely with infrastructure-related components, their role in shaping effective and responsive health security systems is critical.

### 3.2 Rankings and clustering results

Table 3 summarizes the aggregated Global Health Security Index (GHSI) performance scores (AS), corresponding rankings (CR), and cluster assignments (CA) for each HIC under the three clustering experiments.

**Table 3.** Aggregated GHSI performance scores, rankings, and cluster assignments for high-income countries across three clustering cases.

Country	Case 1			Case 2			Case 3		
	AS	CR	CA	AS	CR	CA	AS	CR	CA
United States of America	76.09	1	1	68.26	1	1	81.59	2	1
Australia	72.16	2	1	63.98	3	1	80.82	3	1
Finland	71.45	3	1	67.30	2	1	79.96	7	1
Canada	68.70	4	1	60.45	9	2	77.65	11	1
Slovenia	68.19	5	1	62.30	5	1	81.74	1	1
United Kingdom	67.74	6	1	57.61	11	1	65.12	29	1
Netherlands	66.18	7	1	62.59	4	1	80.40	5	1
Denmark	65.82	8	1	61.42	7	1	79.33	8	1
South Korea	65.65	9	1	56.48	12	1	67.34	22	1
Sweden	65.62	10	1	60.20	10	2	80.48	4	1
Germany	65.58	11	1	60.57	8	1	78.72	10	1
Latvia	60.88	13	1	55.74	15	1	67.08	23	1
Belgium	60.60	16	1	55.91	14	1	76.15	14	2
Japan	59.63	17	1	56.10	13	1	70.82	16	2
New Zealand	59.13	19	1	62.25	6	2	76.28	13	2
Lithuania	57.18	20	1	53.82	16	2	70.77	17	2
France	62.26	12	2	51.71	20	1	73.76	15	2
Norway	60.77	14	2	53.56	18	1	80.04	6	2
Spain	60.66	15	2	52.73	19	1	79.26	9	1
Switzerland	59.62	18	2	49.12	25	1	76.94	12	2
Austria	57.15	21	2	47.77	31	2	69.40	18	2
Portugal	56.71	22	2	53.75	17	1	68.07	20	2
Singapore	56.64	23	2	49.08	26	1	64.34	30	2
Estonia	55.53	24	2	47.77	32	1	65.15	28	2
Ireland	55.20	25	2	43.88	37	2	65.80	25	2
Poland	55.02	26	2	51.24	21	1	62.19	31	2
Hungary	54.69	27	2	48.34	28	1	66.00	24	2
Chile	54.59	28	2	50.61	23	1	65.66	27	2
Czech Republic	53.87	29	2	48.06	30	1	65.68	26	2
Slovakia	53.24	30	2	49.33	24	2	61.41	32	2
Italy	51.89	31	2	48.13	29	1	59.92	36	2
Greece	51.05	32	2	44.45	35	1	69.12	19	2
Croatia	49.29	33	2	48.84	27	2	60.50	34	2
Israel	48.92	34	2	41.72	39	1	60.16	35	2
Luxembourg	48.47	35	2	42.01	38	1	67.64	21	3
Qatar	46.89	37	2	47.65	33	3	58.11	37	3
Iceland	48.00	36	3	50.92	22	3	52.99	38	3
Liechtenstein	45.71	38	3	41.54	40	3	61.41	33	3
Saudi Arabia	44.98	39	3	47.61	34	3	45.64	49	3
Cyprus	42.07	40	3	39.57	47	2	51.62	40	3
Oman	40.01	41	3	38.80	49	3	43.80	52	3
United Arab Emirates	39.83	42	3	40.68	42	4	47.43	44	3
Malta	39.73	43	3	39.80	46	3	52.59	39	3
Uruguay	39.71	44	3	37.87	50	3	45.40	51	3
Kuwait	38.49	45	3	35.70	53	3	42.93	54	3
Brunei	38.24	46	3	44.43	36	4	46.88	45	3
Bahrain	37.57	47	3	41.25	41	3	37.74	56	3
Trinidad and Tobago	37.28	48	3	40.18	43	3	46.84	46	3
Barbados	33.59	49	3	40.03	44	4	49.51	42	4
Monaco	33.53	50	3	33.78	56	3	50.64	41	3
Andorra	32.58	51	3	34.27	55	4	45.84	48	4
San Marino	32.56	52	3	34.99	54	3	48.44	43	3
Seychelles	32.53	53	3	37.20	51	4	39.95	55	4
St Kitts & Nevis	31.27	54	3	39.82	45	4	45.46	50	4
Antigua & Barbuda	30.08	55	3	36.18	52	4	46.40	47	4
Bahamas	29.87	56	3	38.84	48	4	43.10	53	4
Palau	22.71	57	4	23.04	57	4	26.20	57	4
Cook Islands	22.50	58	4	22.61	58	4	20.85	58	4
Nauru	18.73	59	4	20.37	59	4	19.39	59	4

Table 3 summarizes the performance scores, rankings, and cluster assignments for each high-income country across the three distinct clustering scenarios.

In Case 1, the average scores were calculated based on grouped indicators with strong factor loadings across the nine principal components extracted via PCA. Case 2 presents the mean performance scores derived from the 13 most influential indicators loaded on the first principal component, representing the dominant dimension of national health security capacity. Case 3 serves as a baseline using aggregated scores from the six original GHSI categories. Each row in Table 4 represents an individual country, listing its performance score, ranking, and assigned cluster for all three cases. Countries were classified into four performance tiers, where Cluster 1 denotes the highest level of capacity (“High Performance”) and Cluster 4 represents the lowest level (“Critical Concern”), indicating severe deficiencies in health security systems.

**Table 4.** Health security performance clusters of the HICs.

Cluster level	Case 1	Case 2	Case 3
Cluster 1	United States of America, Australia, Finland, Canada, Slovenia, United Kingdom, Netherlands, Denmark, South Korea, Sweden, Germany, Latvia, Belgium, Japan, New Zealand, Lithuania	United States of America, Australia, Finland, Slovenia, United Kingdom, Netherlands, Denmark, South Korea, Sweden, Germany, Latvia, Belgium, Japan, France, Norway, Spain, Poland, Hungary, Chile, Czech Republic, Switzerland, Portugal, Singapore, Estonia, Italy, Greece, Israel	United States of America, Australia, Finland, Canada, Slovenia, United Kingdom, Netherlands, Denmark, South Korea, Sweden, Germany, Latvia, Spain
Cluster 2	France, Norway, Spain, Switzerland, Austria, Portugal, Singapore, Estonia, Ireland, Poland, Hungary, Chile, Czech Republic, Slovakia, Italy, Greece, Croatia, Israel, Luxembourg, Qatar	Canada, Sweden, New Zealand, Lithuania, Austria, Ireland, Slovakia, Croatia, Cyprus	Belgium, Japan, New Zealand, Lithuania, France, Norway, Switzerland, Austria, Portugal, Singapore, Estonia, Ireland, Poland, Hungary, Chile, Czech Republic, Slovakia, Italy, Greece, Croatia, Israel, Luxembourg, Qatar
Cluster 3	Iceland, Liechtenstein, Saudi Arabia, Cyprus, Oman, United Arab Emirates, Malta, Uruguay, Kuwait, Brunei, Bahrain, Trinidad and Tobago, Barbados, Monaco, Andorra, San Marino, Seychelles, St Kitts & Nevis, Antigua & Barbuda, Bahamas	Qatar, Iceland, Liechtenstein, Saudi Arabia,Oman, Malta, Uruguay, Kuwait, Bahrain, Trinidad and Tobago, Monaco, San Marino	Luxembourg, Qatar, Iceland, Liechtenstein, Saudi Arabia, Cyprus, Oman, United Arab Emirates, Malta, Uruguay, Kuwait, Brunei, Bahrain, Trinidad and Tobago, Monaco, San Marino, Seychelles, St Kitts & Nevis, Antigua & Barbuda, Bahamas
Cluster 4	Palau, Cook Islands, Nauru	United Arab Emirates, Brunei,Barbados, Andorra, Seychelles, St Kitts & Nevis, Antigua & Barbuda, Bahamas, Palau, Cook Islands, Nauru	Barbados, Andorra, Seychelles, St Kitts & Nevis, Antigua & Barbuda, Bahamas, Palau, Cook Islands, Nauru

**Figure 2.**
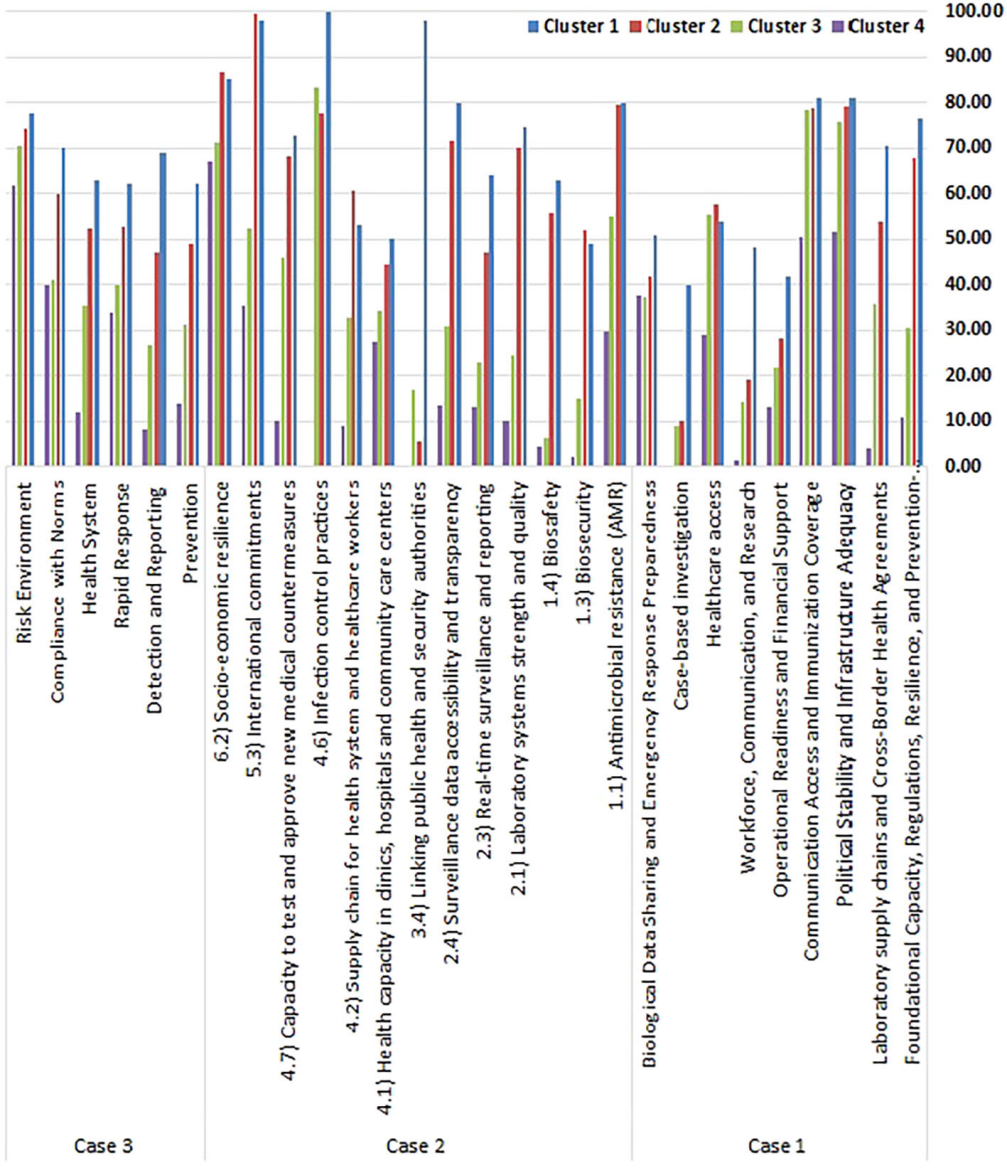
Average health security performance scores (Centroid Values) across the clusters. Figure 2 shows the average performance scores for each cluster across the three different clustering cases, highlighting the distinct profiles of each group.

Table 5 and Figure 2 display the centroid values for each cluster across the three clustering cases, providing a quantitative overview of the average performance profile for each group. These centroid values help define the strengths and weaknesses of countries in each performance tier.

**Table 5.** Average health security performance scores (Centroid Values) across the clusters.

Case	Factor, indicator, or domain	Cluster 1	Cluster 2	Cluster 3	Cluster 4
Case 1	Foundational Capacity, Regulations, Resilience, and Prevention-Detection Systems	76.43	67.83	30.27	10.71
	Laboratory supply chains and Cross-Border Health Agreements	70.31	53.75	35.63	4.17
	Political Stability and Infrastructure Adequacy	80.93	79.29	75.80	51.56
	Communication Access and Immunization Coverage	80.89	78.77	78.23	50.57
	Operational Readiness and Financial Support	41.79	28.22	21.66	13.18
	Workforce, Communication, and Research	48.18	19.10	14.17	1.39
	Healthcare access	53.69	57.69	55.31	29.00
	Case-based investigation	39.84	10.00	8.75	0.00
	Biological Data Sharing and Emergency Response Preparedness	50.73	41.73	37.26	37.52
Case 2	1.1) Antimicrobial resistance (AMR)	80.08	79.63	54.85	29.54
	1.3) Biosecurity	48.94	51.79	15.00	2.00
	1.4) Biosafety	62.96	55.56	6.25	4.55
	2.1) Laboratory systems strength and quality	74.54	70.14	24.48	10.23
	2.3) Real-time surveillance and reporting	63.89	47.22	22.92	13.07
	2.4) Surveillance data accessibility and transparency	80.06	71.48	30.81	13.47
	3.4) Linking public health and security authorities	98.15	5.56	16.67	0.00
	4.1) Health capacity in clinics, hospitals and community care centers	50.07	44.56	34.36	27.23
	4.2) Supply chain for health system and healthcare workers	52.97	60.48	32.87	9.10
	4.6) Infection control practices	100.00	77.78	83.33	0.00
	4.7) Capacity to test and approve new medical countermeasures	72.69	68.06	45.83	10.23
	5.3) International commitments	97.87	99.66	52.35	35.23
	6.2) Socio-economic resilience	85.36	86.53	71.06	67.12
Case 3	Prevention	62.09	48.86	31.37	13.79
	Detection and Reporting	69.08	46.99	26.68	8.25
	Rapid Response	62.00	52.66	39.95	33.89
	Health System	63.06	52.44	35.29	11.95
	Compliance with Norms	70.03	59.96	41.13	39.73
	Risk Environment	77.46	74.23	70.47	61.62

Table 5 displays the centroid values for each cluster, offering a quantitative overview of the average performance profile of each group.

In Case 1, which utilized scores based on the nine extracted components, clustering revealed clear stratification of national health security capacities. The average health security performance scores (centroid values) across the four clusters, based on nine composite factors, illustrate clear disparities in preparedness and capacity among high-income countries. Cluster 1 stands out as the most robust group, consistently achieving the highest scores across nearly all domains. It demonstrates strong foundational systems (76.43), advanced laboratory and cross-border agreements (70.31), and high political stability and infrastructure adequacy (80.93). Moreover, communication and immunization coverage (80.89) and healthcare access (53.69) are well-established, while operational readiness (41.79), workforce and research (48.18), case-based investigation (39.84), and biological data sharing and emergency preparedness (50.73) also reflect relatively strong capacities.

Cluster 2 shows a solid, though slightly reduced, performance with high scores in political stability (79.29) and communication (78.77), but noticeable declines in workforce and research (19.10), case-based investigation (10.00), and operational readiness (28.22), indicating weaknesses in emergency implementation and investigative systems.

Cluster 3 displays moderate to low performance across most factors, with only political stability (75.80) and communication coverage (78.23) maintaining high levels, while foundational systems (30.27), operational readiness (21.66), and biological preparedness (37.26) remain underdeveloped.

Cluster 4 is the weakest group, with critically low scores across almost all domains, including foundational systems (10.71), laboratory capacity (4.17), and case-based investigation (0.00). Despite slightly better performance in biological preparedness (37.52), it still lags far behind the other clusters.

These patterns highlight distinct health security profiles: Cluster 1 countries are comprehensively prepared, Cluster 2 has structural strength but operational gaps, Cluster 3 shows selective resilience, and Cluster 4 reflects systemic vulnerability and limited capacity to respond effectively to health threats.

In Case 2, clustering was based on the 13 key indicators loaded on the dominant principal component. The analysis revealed four tiers of performances. Cluster 1 was the highest-performing group, excelling in infection control (100), public health-security integration (98.15), and international commitments (97.87). Cluster 2 reflected moderate-to-high performance, with strengths in AMR control (79.63), socio-economic resilience (86.53), and laboratory quality (70.14), but exhibited a pronounced weakness in inter-agency coordination (5.56). Cluster 3 displayed mixed results, with relatively strong scores in infection control (83.33) and socioeconomic resilience (71.06), but significant weaknesses in biosecurity (15) and surveillance systems. In contrast, Cluster 4 revealed severe capacity gaps across most indicators, including infection control (0), biosecurity (2), and testing capacity (10.23), underscoring critical shortfalls even among resource-rich countries.

In Case 3, using the six original GHSI categories, the countries were again divided into four performance clusters. Cluster 1 emerged as the top-performing group, with consistently strong scores across all domains—detection and reporting (69.08), prevention (62.09), and risk environment management (77.46) being particularly notable. Cluster 2 showed moderately high performance, excelling in compliance with international norms (59.96) and risk governance (74.23), but needing improvement in early detection (46.99) and prevention (48.86). Cluster 3 reflected moderate capabilities, performing relatively well in environmental risk resilience (70.47), but lagging in detection systems (26.68) and health infrastructure (35.29). Cluster 4 was the lowest-performing group, with critically low scores in prevention (13.79), detection (8.25), and health system strength (11.95), indicating serious gaps in foundational preparedness.

Together, these clustering results reveal consistent stratifications in health security performance among HICs, emphasizing persistent disparities in capacity, coordination, and systemic readiness, even in economically advanced settings. The multi-scenario analysis enhances the robustness of the findings and provides a comprehensive framework for comparative health security assessments and policy prioritization.

## 4. Discussion

### 4.1 Uncovering foundational health security dimensions: Insights from Principal Component Analysis

The Principal Component Analysis (PCA) in Table 1 shows nine different hidden factors that together explain about 74.50% of the total variation in health security indicators among high-income countries (HICs). According to the Kaiser criterion, these components are significant as their eigenvalues exceed 1.0. ^51^ This dimensionality reduction is crucial for uncovering the underlying structures that shape the national health security profiles. Notably, components 1–3 are pivotal in understanding the data, contributing 51.81% of the explained variance. Consequently, components with eigenvalues greater than one were retained for further analysis.

In the scree plot shown in Figure 2, a distinct “elbow” is visible after the ninth component. This clear visual cue provides compelling evidence that only these nine components contain significant and relevant information. The presence of the elbow indicates a point of diminishing returns, suggesting that additional components beyond the ninth contribute relatively little to the overall variance explained by the model. This method aligns with well-established practices in principal component analysis (PCA), which aims to identify and isolate the most important dimensions within complex datasets. Such approaches are essential for effective data reduction and interpretation, as highlighted by Gewers et al. ^49^

Furthermore, the rotated component matrix and the variable loadings in Table 2 clearly demonstrate that Component 1 encompasses a comprehensive array of capacities that are vital for national health security. This component consolidates variables related to foundational systems, including prevention, detection, response, and resilience, which are crucial for establishing robust public health systems, particularly in high-income countries (HICs). High-loading variables include prevention-related indicators such as antimicrobial resistance (AMR), biosecurity, and biosafety, along with detection and reporting measures such as laboratory system strength and quality, real-time surveillance and reporting, and surveillance data accessibility and transparency. Rapid response capacities, such as linking public health and security authorities, and systemic readiness indicators, such as health capacity in clinics, hospitals, and community care centers; healthcare supply chains; infection control practices; and medical countermeasure approval capacity, are also prominent. Furthermore, overarching elements such as international commitments and socio-economic resilience are heavily represented. Collectively, these variables underscore the structural backbone of health security in HICs and confirm the multidimensional nature of public health preparedness. Among the most critical dimensions within this component are prevention and detection capacities, which are foundational to early warning systems. Numerous studies have emphasized the role of AMR surveillance in tracking resistance trends and supporting evidence-based medical decisions. ^55^ The integration of human, animal, and environmental health—the so-called “One Health” approach—is vital for understanding AMR dynamics and reducing its spread. Genomic surveillance strengthens this approach by enabling the real-time tracking of pathogen mutations and transmission pathways, thereby improving response strategies. Global frameworks, such as the WHO’s International Health Regulations (IHR), place a strong emphasis on biosafety and biosecurity as core tools for managing biological risks and deterring bioterrorism. ^56^ A culture of institutional responsibility, combined with strong legislation, is essential for biosafety governance and for responding to the ethical and technical challenges posed by advances in biotechnology. ^57^

However, despite their recognized importance, many HICs perform poorly in these areas, ^38^ with 46% and 44% scoring below the acceptable thresholds in biosecurity and biosafety, respectively. Such gaps limit their capacity to detect, contain, and respond to biological threats, thereby making them susceptible to future crises. Addressing these weaknesses will require targeted investments in policy reform, laboratory oversight, professional training, and expanded international cooperation on genomic and AMR surveillance.

Closely linked to the issue of prevention is the effectiveness of surveillance and reporting systems that facilitate the rapid identification and assessment of health threats. Laboratory systems serve as the diagnostic backbone of outbreak response, enabling authorities to confirm cases and trace the transmission routes. ^58^ When integrated with real-time data systems, such as electronic health records (EHRs), these laboratories aid in the rapid detection of epidemiological trends and unusual patterns. ^59^ The transparency and accessibility of surveillance data further enhance global cooperation, assisting governments and international organizations in responding to them collectively. ^60^ However, deficiencies remain prevalent. As shown by, ^38^ 34% of HICs scored below 40% for Laboratory Systems Strength and Quality, 47% fell short in Real-Time Surveillance and Reporting, and 31% underperformed in Surveillance Data Accessibility and Transparency. These shortcomings delay outbreak detection and intervention, increasing the risk of disease spread and eroding public trust in the health systems. To address these issues, HICs must modernize their surveillance architecture by integrating digital health systems, fostering cross-sectoral data exchange, investing in epidemiological workforce development, and enhancing coordination between the health and security sectors. ^61^

Equally important to Component 1 is the relationship between health system capacity and supply chain management, both of which are essential for operational readiness in the health sector. Health infrastructure in clinics, hospitals, and community centers must be robust and scalable to handle both routine care and emergency cases. The COVID-19 pandemic underscored this necessity, revealing a significant strain on health systems during periods of peak demand. ^62^

Simultaneously, the availability and distribution of critical supplies, such as pharmaceuticals, personal protective equipment, and testing kits, depend on the integrity of the health system supply chains. Disruptions caused by geopolitical crises, pandemics, or natural disasters can severely impair these supply systems and jeopardize response efforts. ^63^ Despite these opportunities, the current performance remains suboptimal. In the Health Security Index, ^38^ 49% of HICs scored below 40% in health capacity in clinics, 42% in hospitals and community care centers, and 37% in healthcare supply chains. These weaknesses compromise service continuity and diminish the crisis response capacity. To address these vulnerabilities, countries should pursue infrastructure upgrades, expand and train the healthcare workforce, digitize supply logistics, and implement national stockpiling strategies that are aligned with real-time demand forecasting systems.

Taken together, the variables captured under Component 1 offer a detailed portrait of the foundational systems underpinning effective health security. However, as evidenced by the widespread deficiencies among HICs, structural gaps remain in prevention, detection, infrastructure, and supply logistics. These gaps not only endanger national resilience but also weaken the global health-security architecture. Moving forward, strategic and sustained investments across these domains, combined with innovation, collaboration, and systems integration, are essential to ensure that high-income countries are better prepared for future health emergencies.

### 4.2 Insights from clustering results

This study employed a multi-case clustering approach to deliver a multidimensional assessment of health security performance among high-income countries (HICs). By integrating PCA-based methodologies (Cases 1 and 2) with a baseline clustering model based on the original Global Health Security Index (GHSI) categories (Case 3), the analysis captured both latent structural dimensions and domain-specific preparedness patterns ( Table 3). The findings reveal consistent strengths among leading countries, expose hidden vulnerabilities in others, and emphasize the influence of methodological design on performance interpretation. These insights underscore the importance of using diverse analytical lenses to comprehensively assess health security and inform targeted investments.

***4.2.1 Cluster profiles based on foundational health security capacities (Case 1), and targeted investment priorities***

A notable finding across all clusters was the consistent identification of weaknesses in critical areas (see Table 5), such as case-based investigation (PCA8), emergency response operations and financing (PCA5), and workforce, communication, and research (PCA6). The latter includes essential elements such as the alignment of the Joint External Evaluation (JEE) and Performance of Veterinary Services (PVS) pathways, effective communication with healthcare workers during public health emergencies, the capacity of the epidemiology workforce, and the promotion of a responsible science culture, including oversight of dual-use research.

These deficiencies underscore the persistent operational and governance challenges that significantly impede effective health security preparedness. ^64– 66^ Strategic investments are crucial to address these gaps.

All countries, regardless of their cluster, should prioritize strengthening case-based investigations, communication, workforce development, and dual-use research. This requires a multifaceted approach, including enhancing epidemiological training, establishing clear and robust emergency communication protocols, and fostering a culture of responsible science with stringent ethical oversight. ^67^ Such measures are essential to ensure that public health responses are effective, safe, and ethically sound. ^67^ Additionally, there must be a strong emphasis on bolstering emergency response operations and securing sustainable financing. ^68^ This involves building rapid response capabilities, implementing coordinated emergency protocols, and establishing long-term funding mechanisms to maintain readiness and resilience against future public health threats. ^69^

Conversely, the analysis also indicates that certain domains, such as political stability and infrastructure adequacy (PCA3) and communication access and immunization coverage (PCA4), show relatively consistent performance across the clusters. This suggests that while foundational institutional frameworks and basic public health services are broadly established, the more specialized technical and operational capacities remain unevenly distributed. This disparity highlights key areas where targeted investment and capacity-building efforts will yield the most significant improvements.

The analysis revealed pronounced disparities between the clusters, particularly between high-performing Cluster 1 and low-performing Cluster 4. The most significant divergence was observed in foundational capacities, where Cluster 1 achieved a robust score of 76.43, in stark contrast to Cluster 4’s score of 10.71. Similar substantial gaps are evident in laboratory supply chains and cross-border health agreements (70.31 versus 4.17), workforce development and communication (48.18 versus 1.39), and case-based investigation, where Cluster 4’s score is virtually zero. Operational readiness and financial support also show a steep decline from the highest to lowest performing clusters.

The distinct patterns within each cluster offer a more detailed understanding of these differences. The 20 countries in Cluster 1 consistently achieved high scores across the first four principal components: Foundational Capacity, Regulations, Resilience, and Prevention-Detection Systems (PCA1); Laboratory Supply Chains and Cross-Border Health Agreements (PCA2); Political Stability and Infrastructure (PCA3); and Communication and Immunization Access (PCA4).

Together, these components accounted for over 57% of the total variance, highlighting the strength of their integrated health systems, which are characterized by resilient infrastructure, effective intersectoral governance, and robust surveillance mechanisms. However, even these top-performing nations showed relative weaknesses in operational readiness (PCA5), workforce and research (PCA6), and case-based investigations (PCA8). This suggests that even the most prepared countries have gaps in these areas, marking them as key investment priorities for future development.

Cluster 2 countries, including France, Norway, Spain, and Switzerland, displayed moderate-to-high scores in the first four principal components, particularly in Political Stability (79.29) and Communication Infrastructure (78.77). However, their scores in Biological Data Sharing and Emergency Response Planning (41.73) and operational domains such as workforce (PCA6) (19.10) and case-based investigation (PCA8) (10.00) were significantly lower. This indicates a structural-operational gap: while the institutional and infrastructural components are well established, their agility and responsiveness remain underdeveloped. Strategic investments for this cluster should align with general recommendations, with an added emphasis on strengthening biological data sharing and emergency response planning. ^70^ Specifically, resources should be directed toward developing and regularly exercising national and cross-sector response plans to ensure adaptability to emerging threats. ^71^ Additionally, these countries should enhance their infrastructure and governance mechanisms to support the timely and transparent sharing of genetic, biological data and specimens. ^56^ This includes establishing clear legal frameworks and robust data platforms and fostering international collaboration protocols to reinforce early warning systems, build global trust, and improve coordinated responses to future public health emergencies. ^72^

Cluster 3 countries, such as Iceland, the UAE, and Malta, scored higher in political stability and public communication access (PCA3 and PCA4) but underperformed in foundational capacities (PCA1:30.27), laboratory supply chains, cross-border health agreements (PCA2:35.65), and operational components (PCA5 and PCA6). This profile suggests that while political will and public engagement capacity may exist, they are not matched by the necessary technical or institutional capabilities to implement them. Strategic investments for this cluster should follow the general recommendations for all clusters and for Cluster 2, with an additional focus on strengthening weaknesses in foundational systems (PCA1) and laboratory infrastructure (PCA2). Investments should be directed toward strengthening public health regulations, enhancing diagnostic infrastructure, securing laboratory supply chains, and establishing cross-border agreements for coordinated emergency responses in both the public and animal health sectors. ^55^

Cluster 4 countries, including Nauru, Palau, and the Cook Islands, demonstrated widespread weaknesses across nearly all PCA components, except for political stability and infrastructure adequacy (PCA3) and communication access and immunization coverage (PCA4), where they performed at an average level (approximately 50 out of 100). These countries lack essential components of a health security system. Strategic investments for this group should align with the recommendations for all other clusters but with a phased implementation strategy. Initially, the focus should be on developing core surveillance infrastructure, laboratory capacity, and a foundational regulatory framework for the same. It is crucial to prioritize strengthening foundational systems such as workforce development, emergency communication protocols, and essential service delivery. For these countries, sustained international support is vital to ensure long-term capacity building and resilience. ^2, 3, 8, 9^

***4.2.2 Cluster profiles based on foundational indicators from PCA1 (Case 2), and targeted investment priorities***

A notable observation across all clusters was the persistence of critical weaknesses in the essential preparedness domains. A consistent pattern of deficiencies emerged in several key areas across the identified clusters of studies, indicating widespread challenges in health security preparedness. Notably, biosecurity scores were generally low, except for Cluster 2, which demonstrated a moderate capacity (51.79) for managing biological risks. This indicates a widespread deficiency in the systems and protocols necessary to prevent, detect, and respond to biological threats in the country. ^7, 23^ Similarly, real-time surveillance and reporting mechanisms were found to be inadequate across most clusters, highlighting a pervasive challenge in terms of early warning and rapid information dissemination in these countries. ^8, 9^ Cluster 1 was the only notable outlier in this regard, exhibiting relatively robust capabilities (63.89). Additionally, the health capacity of clinics, hospitals, and community care centers is consistently low. Although Cluster 2 performed slightly better in this area (50.07), the overall trend indicates systemic limitations in the operational readiness and resilience of frontline healthcare services in all three clusters.

Furthermore, one of the most concerning deficiencies identified across the clusters was the notably poor integration between public health and security authorities. With most clusters scoring below 17 out of 100 in this area, the analysis highlights a significant gap in intersectoral coordination, which is crucial for an effective and cohesive response to health emergencies. In this regard, Cluster 1 significantly outperformed Cluster 4, achieving a score more than five times higher.

Conversely, the analysis revealed areas with consistent strength. A key commonality across all clusters was the relatively high performance in socioeconomic resilience, suggesting that the foundational elements of societal stability and preparedness are generally well established, even among clusters with weaker technical capacities. This inherent resilience may serve as a crucial platform for strengthening broader health security frameworks. ^73^ Additionally, antimicrobial resistance (AMR) management emerged as a relative strength in most clusters, indicating a baseline level of institutional and infrastructural capacity to address this challenge. Cluster 4, however, was a notable exception, demonstrating markedly weaker performance in AMR management.

Moving to the cluster-specific results, clusters 1 and 2 display remarkably similar performance profiles, marked by strong health and security capacities. Both clusters demonstrated strong performance in antimicrobial resistance (AMR) management, socioeconomic resilience, and adherence to international commitments. This highlights their well-established structural preparedness, active engagement on a global scale, and supportive sociopolitical environments, all of which contribute to effective health security systems. These strengths are further reinforced by well-established laboratory systems, high accessibility of surveillance data, and a strong capacity to test and approve new medical countermeasures, all of which are indicative of mature diagnostic and regulatory infrastructure.

Despite these strengths, both clusters exhibited moderate biosecurity and biosafety performance, suggesting ongoing vulnerabilities in biological risk management. ^2, 8^ Their healthcare capacities in clinical and community settings are also limited, reflecting constraints on frontline operational preparedness. ^9, 23^

The most significant divergence between the two clusters is public health security coordination. Cluster 1 demonstrates exceptional integration in this area (98.15), indicating highly effective intersectoral coordination mechanisms. In stark contrast, Cluster 2 shows minimal integration (5.56), highlighting a critical gap. Additionally, Cluster 2 lagged behind Cluster 1 in real-time surveillance (47.22 vs. 63.89), indicating weaker early detection and information systems. In short, although both clusters have strong structures, their differences highlight the crucial need for better teamwork across sectors and strong early warning systems to ensure complete health security. ^7, 8^

In contrast, Clusters 3 and 4 demonstrate a similar pattern of widespread underperformance, highlighting significant limitations in nearly all dimensions of health security. Both clusters exhibit profound systemic weaknesses in core technical capacities, especially in biosecurity, biosafety, and laboratory systems. The extremely low scores in these areas reflect a severe lack of essential infrastructure and institutional preparedness for biological risk management and diagnostics. A critical shared challenge is the lack of surveillance capacity. Both real-time surveillance and reporting and surveillance data accessibility remain weak, indicating fragmented information systems and the absence of reliable and early warning mechanisms.

Compounding this issue is the near-total absence of coordinated multisectoral governance, as evidenced by extremely poor scores linking public health and security authorities—an essential pillar of public health emergency management. In terms of health system infrastructure, Clusters 3 and 4 exhibited significant limitations in healthcare service capacity and supply chains for healthcare workers and resources, further weakening operational resilience. Their ability to test and approve new medical countermeasures is similarly constrained, limiting their capacity for timely innovation and response during crises. ^55^

Despite their pervasive underperformance, both clusters exhibit notable strength in socioeconomic resilience, indicating a foundational level of stability that can be utilized for future capacity development.

Furthermore, a notable point of differentiation between the two is observed in infection control practices, where Cluster 3 shows moderate performance (83.33), unlike Cluster 4, which records a score of zero. Nonetheless, both fall short of international commitments, reflecting weak alignment with global health frameworks and limited participation in cooperative initiatives. ^3, 7, 9^

In summary, the clustering results highlight the polarized landscape of global health security. Clusters 1 and 2 demonstrate relatively advanced health security systems; however, significant structural gaps persist, particularly in bio-risk governance and intersectoral coordination. In contrast, Clusters 3 and 4 encountered deep and widespread vulnerabilities, notably in technical capacity, surveillance infrastructure, and cross-sectoral integration. These common deficiencies—especially in biosecurity, biosafety, surveillance systems, and public health-security coordination—pose substantial risks to effective preparedness and response. To bolster resilience, countries in lower-performing clusters urgently need foundational capacity building, institutional reform, and sustained international support. Simultaneously, even higher-performing countries must address their weakest links to achieve truly comprehensive and equitable global health security.

### 4.3 The comparison between clustering outcomes: Methodological influence, country-specific shifts, and strategic investment implications

Comparing the clustering results from Case 1, which relies on nine PCA components, with those from Case 2, which emphasizes 13 key indicators from the first principal component (PCA1), underscores how methodological focus can shape a country’s rankings and investment priorities. Case 1 provides a comprehensive, system-wide evaluation of health security performance, whereas Case 2 focuses on core structural resilience.

For example, Canada falls from rank 4 in Case 1 to rank 9 in Case 2, revealing foundational technical weaknesses that are not fully captured in the broader assessment. In contrast, New Zealand significantly improved its rank from 19 to 6 but remained in Cluster 2, indicating strong foundational infrastructure but some limitations in overall system capacity.

Additionally, countries such as France, Norway, and Spain shifted from Cluster 2 in Case 1 to Cluster 1 in Case 2, highlighting strong foundational bases that are less evident in the multidimensional analysis. These variations have clear policy implications: countries consistently ranked in Cluster 1, such as the USA and Finland, should maintain their leadership through innovation and system integration; those that decline in Case 2, such as Canada and Sweden, need to bolster their technical and foundational capacities; those that improve, including France and Spain, should focus on developing their operational systems to align with their structural strengths; and countries occupying lower clusters in both cases, such as Nauru and Palau, require comprehensive foundational investments to build basic health security capabilities.

The third clustering approach (Case 3), which employs the original six equally weighted GHSI categories, serves as a useful baseline but lacks sensitivity to the key performance drivers and fails to differentiate between foundational and operational strengths of the countries. For instance, France and Norway are undervalued in Case 3 compared to their positions in PCA-based clusters, whereas Canada and Sweden, despite being highly ranked in both Case 3 and Case 1, perform worse in Case 2, highlighting critical foundational gaps. Thus, PCA-based clustering offers greater diagnostic clarity: Case 1 supports broad, system-wide reforms, whereas Case 2 focuses on technical and foundational investments. The GHSI categorical framework can complement these insights by providing thematic perspectives but should not be used in isolation for investment planning. ^3, 9, 23^ Together, these methods offer a more comprehensive and nuanced basis for prioritizing health security improvement.

From an alternative perspective, synthesizing results across various clustering cases indicates that in all clustering methodologies, certain core high-performing nations, such as the United States, Australia, Finland, and the Netherlands, consistently achieved top rankings and cluster assignments. This consistency reflects the robustness of their health security systems. However, countries such as Canada and Sweden exhibit variability depending on the analytical method used, revealing hidden vulnerabilities that aggregate scores might obscure. For example, Luxembourg, often seen as well-resourced, falls into lower-performing clusters in GHSI-based Case 3, suggesting overlooked structural weaknesses. Small island nations and microstates—including Nauru, Palau, and the Cook Islands—consistently rank lowest across all cases, underscoring persistent, deep-rooted structural barriers that necessitate sustained international assistance and regional cooperation to build a minimum functional capacity. Integrating insights from PCA-driven clustering methods with the original GHSI categorical framework offers the clearest and most nuanced understanding of each country’s health security strengths and weaknesses. This triangulation enables the identification of priority areas for targeted investments and policy interventions.

Those, this study highlights how the choice of methodological design profoundly influences the interpretation of health security performance. PCA effectively identifies the most influential variables, providing deeper insights into systemic readiness, whereas clustering based on the GHSI captures a broader thematic overview. Together, these approaches form a robust and complementary framework for analysis. By employing multi-method clustering, policymakers can cross-validate findings, design tiered investment strategies, and address both structural and operational gaps, ultimately ensuring that health preparedness systems are not only theoretically strong but also functionally effective during crises.

### 4.4 Comparative Analysis of Clustering Outcomes: Proposed Model vs. MCDM-Based Models

In this analysis, we compared the clustering results of the second case of our proposed model with those of nine established k-means clustering models, each of which utilized a different multi-criteria decision-making (MCDM) approach. Our model stands out by clustering countries using the K-means method based on a comprehensive matrix of 13 indicators without assigning weights to them. In contrast, the nine comparison models cluster countries based on single-indicator scores derived from the ranking outputs of the MCDM methods, with and without weighting. We employed combinations of CoCoSo, TOPSIS, and VIKOR MCDM methods with Equal Weighting (EW), Entropy, and D-CRITIC weighting methods. ^2, 3, 8, 9^

Table 6 summarizes the results of the nine models as follows: EW–CoCoSo (M1), Entropy–CoCoSo (M2), D-CRITIC–CoCoSo (M3), EW–TOPSIS (M4), Entropy–TOPSIS (M5), D-CRITIC–TOPSIS (M6), EW–VIKOR (M7), Entropy–VIKOR (M8), and D-CRITIC–VIKOR (M9), alongside the outcomes of the proposed model (PM).

**Table 6.** Clustering Outcomes, correlation and cluster shifts in comparative Models.

Country	CoCoSo-based models			TOPSIS-based models			VIKOR-based models			PM
	M1	M2	M3	M4	M5	M6	M7	M8	M9	
Andorra	4	4	4	4	4	4	4	4	4	4
Antigua & Barbuda	4	4	4	4	4	4	4	4	4	4
Australia	1	1	1	1	1	1	1	1	1	1
Austria	2	2	1	2	3	2	3	3	3	2
Bahamas	4	4	4	4	4	4	4	4	4	4
Bahrain	3	3	3	3	4	3	4	4	4	3
Barbados	4	4	4	4	4	4	4	4	4	4
Belgium	1	1	1	1	1	1	1	1	1	1
Brunei	3	3	3	3	4	3	4	4	4	4
Canada	2	2	1	1	2	2	3	3	3	2
Chile	2	1	1	1	1	1	2	2	1	1
Cook Islands	4	4	4	4	4	4	4	4	4	4
Croatia	2	2	2	2	3	2	3	3	3	2
Cyprus	3	3	2	3	3	3	3	4	4	2
Czech Republic	2	1	1	1	1	1	1	2	1	1
Denmark	1	1	1	1	1	1	1	1	1	1
Estonia	2	1	1	1	1	1	2	2	1	1
Finland	1	1	1	1	1	1	1	1	1	1
France	1	1	1	1	1	1	1	1	1	1
Germany	1	1	1	1	1	1	2	2	1	1
Greece	2	1	1	1	1	1	2	2	2	1
Hungary	2	1	1	1	1	1	1	2	1	1
Iceland	3	3	2	3	4	3	3	4	4	3
Ireland	2	2	1	1	3	2	3	3	3	2
Israel	2	2	1	2	2	2	2	2	2	1
Italy	2	1	1	1	1	1	2	2	1	1
Japan	2	1	1	1	2	1	2	2	1	1
Kuwait	3	2	2	3	2	2	3	3	3	3
Latvia	1	1	1	1	1	1	1	1	1	1
Liechtenstein	3	3	2	3	4	3	4	4	4	3
Lithuania	2	2	1	2	3	2	3	3	2	2
Luxembourg	2	2	1	2	2	2	2	2	2	1
Malta	3	3	3	3	4	3	3	4	4	3
Monaco	3	3	3	3	4	3	4	4	4	3
Nauru	4	4	4	4	4	4	4	4	4	4
Netherlands	1	1	1	1	1	1	1	1	1	1
New Zealand	2	2	2	2	3	3	3	3	4	2
Norway	2	1	1	1	1	1	1	2	1	1
Oman	3	3	3	3	4	3	3	4	4	3
Palau	4	4	4	4	4	4	4	4	4	4
Poland	2	2	1	2	2	1	2	2	2	1
Portugal	2	2	1	1	2	2	1	2	2	1
Qatar	3	2	2	3	2	2	3	3	2	3
San Marino	4	3	3	4	4	3	4	4	4	3
Saudi Arabia	3	3	2	3	3	3	3	4	4	3
Seychelles	4	4	4	4	4	4	4	4	4	4
Singapore	1	1	1	1	1	1	2	1	1	1
Slovakia	2	2	1	2	3	2	3	3	3	2
Slovenia	1	1	1	1	1	1	2	1	2	1
South Korea	1	1	1	1	1	1	1	1	1	1
Spain	1	1	1	1	1	1	1	1	1	1
St Kitts & Nevis	4	4	4	4	4	4	4	4	4	4
Sweden	1	2	1	1	2	2	3	3	3	2
Switzerland	2	2	1	2	2	2	2	2	2	1
Trinidad and Tobago	4	3	3	4	4	3	4	4	4	3
United Arab Emirates	3	3	3	3	4	3	4	4	4	4
United Kingdom	1	1	1	1	1	1	1	1	1	1
United States of America	1	1	1	1	1	1	1	1	1	1
Uruguay	3	3	3	3	4	3	4	4	4	3
**Cor.**	0.90	0.94	0.94	0.93	0.91	0.94	0.91	0.90	0.89	1
**T. shifts**	19	10	13	12	24	10	27	33	26	0

The table also reports the correlations (Cor.) between our model and the nine comparison models, as well as the total number of countries that experienced a cluster shift when compared with our model (T. shifts). As shown in Table 6, the high correlation values indicate a strong alignment between our model and the comparison models. Notably, the highest correlation (0.94) was found for the Entropy-CoCoSo (M2), D-CRITIC-CoCoSo (M3), and D-CRITIC-TOPSIS (M6) models, suggesting that their clustering outcomes closely resembled those of our models. This reinforces the validity of our model, as it effectively captures similar grouping patterns based on a diverse range of indicators.

A significant aspect of our comparison is the number of countries that shifted their clusters by one level when comparing our model to each of the nine models. The total number of countries that experienced a shift is presented in table. The highest number of shifts occurred in the Entropy-VIKOR model (M8) (33 countries), indicating substantial differences in clustering outcomes. In contrast, the Entropy-CoCoSo (M2), D-CRITIC-CoCoSo (M3), and D-CRITIC-TOPSIS (M6) models resulted in the fewest shifts (10, 13, and 10 countries, respectively), suggesting a closer alignment with our model’s clustering. These results highlight that while our model aligns closely with established models in terms of correlation, there are notable differences in how countries are clustered in the two models.

The variations in the number of countries shifting clusters underscore the impact of using a comprehensive indicator matrix rather than relying solely on single indicator scores. Our approach provides a more nuanced understanding of country groupings by incorporating multiple performance dimensions, whereas the comparison models may oversimplify clustering by focusing on singular aspects. This complexity is particularly beneficial for policymakers and researchers seeking to understand the multifaceted nature of the indicators influencing country rankings. In conclusion, the robust correlation between our model and the existing ones, combined with the substantial number of cluster shifts, suggests that while our method is consistent with established approaches, it also offers a distinct perspective that can lead to different interpretations of country clustering.

### 4.5 Implication of study

***4.5.1 General implications of study***

The findings of this study have significant implications for global health policy, particularly for HICs and international organizations. Utilizing PCA and a multi-scenario clustering approach, this study provides a detailed understanding of health security, going beyond simple aggregated assessments. This highlights the complex interactions between foundational capacities, operational readiness, and systemic vulnerabilities. The emergence of distinct performance clusters among HICs clearly shows that economic prosperity alone does not ensure strong health and security. Instead, it requires strategic coherence, wise investment, and a steadfast commitment to continuous adaptation.

First, this study acts as a sophisticated diagnostic tool for policymakers. Detailed insights into the strengths and weaknesses of various HIC clusters, ranging from comprehensively prepared nations to those facing critical foundational gaps, aid in developing highly tailored interventions. For example, countries in Cluster 1, despite their commendable overall performance, show relative weaknesses in terms of operational readiness and workforce development. This indicates a strategic need to shift the policy focus from broad capacity building to enhancing agility and rapid mobilization and cultivating specialized human capital in epidemiology and emergency response. Conversely, nations in lower-performing clusters, such as Nauru, Palau, and the Cook Islands, urgently need fundamental and phased investments in basic surveillance, diagnostic capabilities, and regulatory frameworks. Such foundational improvements often require sustained international assistance and collaborative efforts.

Second, this study emphasizes the critical importance of methodological rigor in assessing health security. The comparative analysis across three distinct clustering cases—PCA-based comprehensive assessment, PCA-based foundational indicator focus, and the original Global Health Security Index (GHSI) categories—clearly demonstrates how diverse analytical lenses can yield profoundly varied insights into a country’s true state of preparedness. This suggests that relying solely on a single aggregated index risks obscuring critical vulnerabilities and misdirecting invaluable investments. Therefore, policymakers are encouraged to adopt a multi-methodological approach, triangulating findings from a range of analytical frameworks to develop a holistic and accurate understanding of the national health security landscape. This integrated perspective is crucial for preventing complacency in seemingly high-performing nations and guiding more effective resource allocation in nations with unrecognized deficiencies.

Finally, this study highlights the essential need for continuous learning and adaptation in the realm of global health security. The inherently dynamic nature of health threats, as vividly exemplified by recent pandemics, mandates that health security systems must continually evolve to effectively respond to emerging challenges. The identified structural gaps, even within resource-rich settings, serve as a clarion call for ongoing policy reform, technological advancement, and intensified international collaborative efforts. These findings serve as a direct call to action for HICs to strengthen their internal resilience, actively engage in global health diplomacy, and robustly support capacity building in less prepared nations. This recognition stems from the fundamental understanding that health security is an interconnected, global public good. A proactive and adaptive approach is not merely beneficial but essential for safeguarding global public health against future crises and cultivating a more equitable and secure environment for all.

***4.5.2 Policy implications***

The study also offers practical guidance for policymakers. High-performing clusters should sustain their leadership by focusing on innovation and system integration, while mid- and lower-tier clusters need targeted investments in foundational systems, intersectional coordination, and emergency operational readiness. For small or structurally constrained countries, international partnerships and resource-sharing mechanisms are crucial to bridging persistent capacity gaps. Crucially, prioritization strategies must be tailored to address the specific weaknesses identified in each cluster, ensuring that investments target the most pressing vulnerabilities rather than adhering to generic capacity-building approaches.

### 4.6 Contributions of this study

This study significantly advances the understanding and practice of global health security, particularly in high-income countries (HICs). It provides novel insights and actionable recommendations in the theoretical, methodological, and practical domains. Theoretical Advancements: From a theoretical standpoint, this research refines the conceptualization of health security by transcending conventional, often aggregated assessments. Through the judicious application of Principal Component Analysis (PCA), this study empirically identifies and extracts the latent dimensions underpinning health security capacities in HICs. This innovative approach demonstrates that health security is not merely an aggregation of its components but is significantly influenced by unique underlying elements such as basic capacity, political stability, communication accessibility, and operational preparedness. This deeper understanding enriches the theoretical framework of health security, offering a more nuanced perspective through which national preparedness and response capabilities can be rigorously analyzed. This highlights that even within seemingly homogeneous groups, such as HICs, complex interdependencies and unique structural components significantly influence overall performance.

Methodological Innovations: This study introduces a robust and innovative multi-case clustering approach that seamlessly integrates PCA with K-means clustering. This integration facilitates a comprehensive and comparative assessment of health security patterns from diverse perspectives. By employing clustering based on nine principal components, 13 key indicators from the leading first principal component, and the six original GHSI categories, this study clearly illustrates the significant impact of methodological decisions on the performance interpretation. This multi-methodological triangulation significantly bolsters the credibility and depth of the findings, offering a valuable framework for future research endeavors aimed at uncovering hidden structures and disparities within complex datasets.

Practical Implications: From a practical perspective, the findings offer invaluable and actionable insights for global health policymakers, stakeholders, and national governments. The precise identification of distinct performance clusters among HICs, coupled with meticulous analyses of their inherent strengths and vulnerabilities, empowers the development of highly targeted policy recommendations and interventions. For instance, the study clearly delineates countries that excel in foundational systems but lag in operational agility, thereby guiding specific investments in workforce development and emergency response mechanisms to enhance it.

Conversely, it identifies nations grappling with pervasive weaknesses across nearly all health security domains, underscoring the urgent need for fundamental, phased capacity building, which often requires sustained international support. By providing a clearer, evidence-based picture of where resources are most critically needed and what types of interventions are most appropriate, this study facilitates more efficient resource allocation and strategic planning, ultimately bolstering national resilience and safeguarding global public health against future infectious disease threats. The emphatic emphasis on the interconnectedness of global health security further reinforces the imperative for robust international partnerships and collaborative efforts to address shared vulnerabilities.

### 4.7 Limitations and future work

Although this study advances the understanding of health security in high-income countries (HICs), it also highlights several limitations that point to promising avenues for future research.

First, the use of 2019 and 2021 GHSI data offers crucial insights from before and during the early stages of the pandemic but fails to consider recent changes. Future studies should incorporate more recent data or additional indices to accurately reflect evolving national capabilities and new threats.

Second, although PCA is effective in reducing dimensionality and uncovering latent structures, its interpretability can be somewhat subjective. Despite employing varimax rotation, future research should investigate alternative rotations or methods to assess the stability and robustness of these results.

Third, focusing solely on HICs restricts generalizability to low- and middle-income countries (LMICs), which encounter unique challenges in healthcare delivery. Applying this approach to LMICs and conducting comparisons across income groups could provide broader insights into global health security disparities and tailored capacity-building strategies.

Fourth, although K-means clustering effectively identifies distinct groups, it assumes that the clusters are spherical and of equal size. Exploring other clustering algorithms, such as hierarchical or model-based methods, and evaluating cluster stability over time may uncover more complex structures and improve interpretability. Finally, this study focused on quantitative indicators. Incorporating qualitative methods, such as case studies or expert interviews, could offer deeper, context-specific insights into the sociopolitical and governance factors affecting health security performance, thereby enriching future policy recommendations.

Fifth, the current study did not use objective weighting techniques to determine the relative importance of factors before clustering, treating all indicators equally in the process. Future research could improve analytical precision by applying objective weighting methods, such as CRITIC, Entropy, or MEREC, to derive data-driven weights that reflect the variability and information content of each indicator. These weights can then be integrated into weighted clustering models to produce more nuanced groupings of countries based on their true performance differentials. Additionally, comparing the stability of clustering and ranking outcomes across different weighting schemes, including hybrid approaches that combine objective and subjective inputs, could provide valuable insights into the robustness of the results and inform the best practices for model selection and implementation.

## 5. Conclusion

This study offers a comprehensive and multifaceted evaluation of health security performance among high-income countries (HICs), moving beyond traditional aggregate assessments to delve into the intricate dynamics underlying national preparedness and responses. By employing Principal Component Analysis (PCA) and a multi-scenario clustering framework, we uncovered the latent structural dimensions of health security and systematically grouped countries based on their alignment with these dimensions. Our findings revealed distinct stratifications in performance, showing that despite their economic advantages and advanced health systems, HICs still display significant variations in core capacities, intersectoral coordination, and systemic readiness. The results reaffirm that health security is inherently multidimensional and shaped by interconnected factors such as foundational infrastructure, regulatory environments, political context, and operational effectiveness. The emergence of a dominant first principal component—encompassing key national functions such as surveillance, biosafety, clinical infrastructure, supply chain reliability, and global engagement—highlights the foundational elements essential for resilient health systems. Moreover, the multi-case clustering approach demonstrates how different analytical perspectives can uncover diverse patterns of strength and vulnerability, offering insights that are often obscured by single-dimensional assessments. This study provides practical value for global health policymakers by delineating distinct performance clusters, each with unique strengths and needs. For countries leading in performance, the focus should be on enhancing agility, workforce flexibility, and advanced epidemiological capacities. Conversely, lower-performing groups require foundational investments in basic surveillance, diagnostics, and regulatory systems, which are often supported by international collaboration. Ultimately, this study underscores the need for continuous learning and a multi-method approach to health security assessment—one that addresses both structural and functional gaps. Although high income confers advantages, it does not equate to comprehensive preparedness. Achieving true health security requires strategic alignment, robust operational capacity, and sustained commitment across all domains of prevention, detection, and response. This study contributes valuable insights for evidence-based decision-making and global efforts to enhance resilience against future public health threats in an increasingly interconnected world.

## Ethics and consent

No Ethical approval or consent needed.

## Declaration of generative AI and AI-assisted technologies in the writing process

During the preparation of this work the author(s) used [Paperpal, Quillbot, and ChatGPT] for language refinement and structure. After using this tools, the author(s) reviewed and edited the content as needed and take(s) full responsibility for the content of the publication.

## Data Availability

The data supporting the findings of this study are publicly available and can be accessed through the following repository (Global Health Security Index, Global Health Security Index Data Model and Report. (2021), at https://ghsindex.org/report-model/). ^38^ ***Figshare:*** Revealing Principal Components, Patterns, and Structural Gaps in Health Security among High-Income Countries. Doi: https://doi.org/10.6084/m9.figshare.29582498.v2 ^39^ *The project contains the following underlying data*: GHSI_PCA_Clustering_Supplementary_Data_HICs.xlsx. All data, and processing results related to this study are presented in this file. This supplementary file provides comprehensive support for the findings and methodology presented in the study. It includes detailed outputs from the Principal Component Analysis (PCA), such as factor loadings, eigenvalues, and the percentage of variance explained, along with a full classification of the 37 Global Health Security Index (GHSI) indicators across the nine identified principal components. Additionally, it contains visualizations and datasets for all three clustering scenarios: one based on countries’ average scores across the nine extracted components, another using the 13 high-loading indicators from the first principal component, and a third based on aggregated scores from the six original GHSI categories. The file also presents the resulting cluster centroids, validation comparisons, and identified performance patterns. All data are integrated into a single Excel-based tool that includes the underlying values used to generate the study’s tables and figures. This supplementary resource serves as a detailed and practical reference to replicate the study’s procedures and validate its results. Comparative Analysis of Clustering Outcomes Proposed Model vs. MCDM-Based Models.xlsx This supplementary file serves as a comprehensive resource to explore the nuances of clustering outcomes and summarizes the clustering results for the proposed model (PM) and the nine MCDM-based models: EW--CoCoSo (M1), Entropy--CoCoSo (M2), D-CRITIC--CoCoSo (M3), EW--TOPSIS (M4), Entropy--TOPSIS (M5), D-CRITIC--TOPSIS (M6), EW--VIKOR (M7), Entropy--VIKOR (M8), and D-CRITIC--VIKOR (M9). It supports the findings presented in Section 4.4 by providing transparent and detailed data behind the analysis. This data are publicly available and can be accessed through the following repository ( https://figshare.com/articles/dataset/Supplementary_Dataset_for_Revealing_Principal_Components_Patterns_and_Structural_Gaps_in_Health_ Security_among_High-Income_Countries_/29582498?file=56322971) and archived via [ https://doi.org/10.6084/m9.figshare.29582498.v2]. ^39^ Data are available under the terms of the Creative Commons Attribution 4.0 International license (CC-BY 4.0).
