# Supplementary material for: Revealing Principal Components, Patterns, and Structural Gaps in Health Security among High-Income Countries: A Comparative Analysis Using PCA and a Multi-Scenario Clustering Approach
Source: F1000Res. 2025 Sep 11;14:769. Originally published 2025 Aug 6. [Version 2] doi: 10.12688/f1000research.168082.2 (PMC12441671; doi:10.12688/f1000research.168082.2)
Supplement: Peer review history

# Reviewer report: Reviewer response for version 2

**DOI:** 10.5256/f1000research.187265.r413365

**Published:** 2025-09-16

**Authors:** Jiansong Zheng

**Affiliations:** 1 The Hong Kong Polytechnic University Department of Applied Social Sciences, Kowloon, Hong Kong

Thank you for your revision, which effectively addressed my academic concerns. I have no further comments. All the best!

Is the work clearly and accurately presented and does it cite the current literature?

Yes

If applicable, is the statistical analysis and its interpretation appropriate?

No

Are all the source data underlying the results available to ensure full reproducibility?

No

Is the study design appropriate and is the work technically sound?

Yes

Are the conclusions drawn adequately supported by the results?

Yes

Are sufficient details of methods and analysis provided to allow replication by others?

Yes

Reviewer Expertise:

Health psychology, cross-clutural psychology, positive psychology

I confirm that I have read this submission and believe that I have an appropriate level of expertise to confirm that it is of an acceptable scientific standard.

# Reviewer report: Reviewer response for version 2

**DOI:** 10.5256/f1000research.187265.r413364

**Published:** 2025-09-16

**Authors:** Prof Ahmed Sultan Al-Hegami

**Affiliations:** 1 Sana'a University, Sana'a, Yemen

We thank the researchers for their efforts in improving the article.We do not have any further comments.

Is the work clearly and accurately presented and does it cite the current literature?

Yes

If applicable, is the statistical analysis and its interpretation appropriate?

Yes

Are all the source data underlying the results available to ensure full reproducibility?

Yes

Is the study design appropriate and is the work technically sound?

Yes

Are the conclusions drawn adequately supported by the results?

Yes

Are sufficient details of methods and analysis provided to allow replication by others?

Yes

Reviewer Expertise:

My research interest includes Artificial Intelligence, Data Mining, Machine Learning, Big Data, Data Science, Intelligent Business ,Temporal Databases, Decision Support Systems, Real Time Systems and Knowledge Discovery in Databases.

I confirm that I have read this submission and believe that I have an appropriate level of expertise to confirm that it is of an acceptable scientific standard.

# Reviewer report: Reviewer response for version 1

**DOI:** 10.5256/f1000research.185240.r403551

**Published:** 2025-08-21

**Authors:** Prof Ahmed Sultan Al-Hegami

**Affiliations:** 1 Sana'a University, Sana'a, Yemen

**Revealing Principal Components, Patterns, and Structural Gaps in Health Security among High-Income Countries: A Comparative Analysis Using PCA and a Multi-Scenario Clustering Approach**

**Authors: **Adel A. Nasser , Mijahed Nasser Aljober, Abed Saif Ahmed Alghawli, and Amani A. K. Elsayed

 Overall Assessment

 This study is well-structured and methodologically rigorous, making a significant contribution to the field of global health security. By integrating Principal Component Analysis (PCA) with multi-scenario K-means clustering, the authors transcend conventional aggregated index analyses, revealing latent structures, performance patterns, and disparities among high-income countries (HICs). This study is distinguished by its clear justification of methodological choices, transparent presentation of analytical steps, and comprehensive comparative discussion across multiple clustering scenarios. Focusing on high-income countries addresses a critical gap in the literature, underscoring that economic wealth does not necessarily equate to uniform preparedness. The inclusion of three clustering cases—based on PCA-derived components, dominant indicators, and the original GHSI categories—adds depth and robustness to the findings, offering actionable insights for policy makers. Nonetheless, a few revisions could further enhance the manuscript: 

 1. The research gap is well-identified; however, explicitly stating the study’s hypotheses or expected outcomes would strengthen the framing.

 2. Methods: While the rationale for focusing on HICs is clear, a brief justification for excluding potential comparator groups (e.g., upper-middle-income countries) would help contextualize the study’s scope.

 3. Results: The results narrative is comprehensive but could benefit from highlighting the most policy-relevant findings earlier, before presenting detailed technical results.

 4. Discussion: The discussion effectively links the findings to existing literature; however, separating “Policy Implications” into its own subheading would enhance the impact for decision-makers.

Is the work clearly and accurately presented and does it cite the current literature?

Yes

If applicable, is the statistical analysis and its interpretation appropriate?

Yes

Are all the source data underlying the results available to ensure full reproducibility?

Yes

Is the study design appropriate and is the work technically sound?

Yes

Are the conclusions drawn adequately supported by the results?

Yes

Are sufficient details of methods and analysis provided to allow replication by others?

Yes

Reviewer Expertise:

My research interest includes Artificial Intelligence, Data Mining, Machine Learning, Big Data, Data Science, Intelligent Business ,Temporal Databases, Decision Support Systems, Real Time Systems and Knowledge Discovery in Databases.

I confirm that I have read this submission and believe that I have an appropriate level of expertise to confirm that it is of an acceptable scientific standard.

# Response

**Published:** 2025-08-28

**Authors:** Adel A. Nasser

**Affiliations:** Information Systems, Not Applicable, Sa'ada, Sa'ada, Yemen

**Responses to Peer Review Report of Prof.  Ahmed Sultan Al-Hegami, Sana'a University, Sana'a, Yemen**

**Dear Prof. Ahmed Sultan Al-Hegami,**

 Thank you for your thoughtful review and for the insightful comments provided on our manuscript. Your feedback has been invaluable in guiding our revisions.

**Comment 1:** The research gap is well-identified; however, explicitly stating the study’s hypotheses or expected outcomes would strengthen the framing.

**Response: **We appreciate this important suggestion. In the revised Introduction, we have now explicitly stated the study’s hypotheses to strengthen the framing and clarify the expected outcomes.

**Comment 2:** Methods: While the rationale for focusing on HICs is clear, a brief justification for excluding potential comparator groups (e.g., upper-middle-income countries) would help contextualize the study’s scope.

**Response:** Thank you for your valuable feedback regarding this section. In the revised Methods section, we now provide an explicit justification for excluding upper-middle-income countries.

**Comment 3**: Results: The results narrative is comprehensive but could benefit from highlighting the most policy-relevant findings earlier, before presenting detailed technical results.

**Response:** Thank you for this insightful recommendation. In the revised Results section, we now introduce a concise narrative at the beginning that highlights the most policy-relevant findings before the detailed technical outcomes are presented.

**Comment 4**: Discussion: The discussion effectively links the findings to existing literature; however, separating “Policy Implications” into its own subheading would enhance the impact for decision-makers.

**Response:** Thank you for this valuable suggestion. In the revised Discussion section, we created a separate “Policy Implications” subsection to strengthen the impact for decision-makers.

 We hope that these responses adequately address your concerns and further improve our manuscript. Thank you again for your valuable insights!

# Reviewer report: Reviewer response for version 1

**DOI:** 10.5256/f1000research.185240.r403553

**Published:** 2025-08-21

**Authors:** Jiansong Zheng

**Affiliations:** 1 The Hong Kong Polytechnic University Department of Applied Social Sciences, Kowloon, Hong Kong

This study develops comprehensive indicators of health security performance among high-income countries. The language is smooth, and the structure is well-organized. The paper provides valuable national-level data on health security for future cross-national studies and demonstrates substantial application value. Before publication, I have several suggestions for improvement.

 First, since you introduce Multi-Criteria Decision-Making (MCDM) models and machine learning approaches with empirical cases, it would be worthwhile to apply these models in your analysis. At a minimum, comparing the results with those obtained through principal component analysis could strengthen the robustness of your findings.

 Second, I recommend adding a dedicated subsection on the sample within your methods section. This should include a description of the specific countries analyzed and the criteria used to classify them as high-income countries.

 Finally, to improve transparency, it would be useful to provide a list of the countries within each cluster, ideally presented in an appendix table.

Is the work clearly and accurately presented and does it cite the current literature?

Yes

If applicable, is the statistical analysis and its interpretation appropriate?

No

Are all the source data underlying the results available to ensure full reproducibility?

No

Is the study design appropriate and is the work technically sound?

Yes

Are the conclusions drawn adequately supported by the results?

Yes

Are sufficient details of methods and analysis provided to allow replication by others?

Yes

Reviewer Expertise:

Health psychology, cross-clutural psychology, population health

I confirm that I have read this submission and believe that I have an appropriate level of expertise to confirm that it is of an acceptable scientific standard, however I have significant reservations, as outlined above.

# Response

**Published:** 2025-08-28

**Authors:** Adel A. Nasser

**Affiliations:** Information Systems, Not Applicable, Sa'ada, Sa'ada, Yemen

**Responses to Peer Review Report of Prof. Jiansong Zheng, The Hong Kong Polytechnic University Department of Applied Social Sciences, Kowloon, Hong Kong**

**Dear Prof. Jiansong Zheng,**

 We sincerely thank the reviewers for their insightful comments and constructive feedback, which have greatly improved the quality and clarity of this manuscript. Below, we address each point in detail and describe the corresponding revisions made

**Comment 1:** Since you introduce Multi-Criteria Decision-Making (MCDM) models and machine learning approaches with empirical cases, it would be worthwhile to apply these models in your analysis. At a minimum, comparing the results with those obtained through principal component analysis could strengthen the robustness of your findings.

**Response:** We fully agree with this suggestion. In the revised manuscript, we have now integrated a comparative analysis using MCDM-based clustering models (CoCoSo, TOPSIS, VIKOR combined with three weighting schemes) and compared their results against PCA-based approaches. This addition demonstrates the robustness of findings across different methodological frameworks and highlights how methodological choice can alter country clustering outcomes

**Comment 2:** I recommend adding a dedicated subsection on the sample within your methods section. This should include a description of the specific countries analyzed and the criteria used to classify them as high-income countries.

**Response:** Thank you for your constructive feedback. In response, we have added a new subsection titled “Sample Description: High-Income Countries Analyzed” within the Methods section. This subsection outlines the World Bank classification criteria used, describes the rationale for selecting HICs, and lists all 59 countries included in the study.

**Comment 3:** To improve transparency, it would be useful to provide a list of the countries within each cluster, ideally presented in an appendix table.

**Response:** We appreciate this excellent suggestion. We have added a new Table (Table 4) presenting the full list of countries within each cluster for all three clustering scenarios (PCA-based, GHSI-based, and MCDM-based).

 We hope that these responses adequately address your concerns and further strengthen our manuscript. Thank you again for your valuable insights!
